# *APOE3* and *APOE4* human astrocytes differentially modulate Alzheimer’s disease pathology and microglial responses in chimeric mice

**DOI:** 10.1016/j.celrep.2026.117803

**Published:** 2026-08-08

**Authors:** Joan Cruz-Sese, Marta Mirón-Alcala, María Alfonso-Triguero, Jon Olalde, Leire Ruiz, Nuria Galbis-Gramage, Lorea Cortes, Laura Escobar, Pranav Preman, An Snellinx, Takashi Saito, Takaomi C. Saido, Laura Saiz-Aúz, Alberto Rábano-Gutiérrez, Ana M. Aransay, Ibai Diez, Julia Tcw, Alison Goate, Bart De Strooper, Elena Alberdi, Amaia M. Arranz

**Affiliations:** 1 Achucarro Basque Center for Neuroscience, Leioa, Spain; 2 Department of Neurosciences, University of the Basque Country (UPV/EHU), Leioa, Spain; 3 VIB Center for Brain & Disease Research, Leuven, Belgium; 4 Laboratory for the Research of Neurodegenerative Diseases, Department of Neurosciences, Leuven Brain Institute (LBI), KU Leuven, Leuven, Belgium; 5 Department of Neuropathology, Graduate School of Medicine, The University of Tokyo, Tokyo, Japan; 6 Laboratory for Proteolytic Neuroscience, RIKEN Center for Brain Science, Wako, Japan; 7 Fundacion CIEN, Madrid, Spain; 8 Center for Cooperative Research in Biosciences (CIC BioGUNE), Science and Technology Park of Bizkaia, 48160 Derio, Spain; 9 Centre for Networked Biomedical Research on Liver and Digestive Diseases (CIBERehd -ISCIII), Madrid, Spain; 10 Computational Neuroimaging Lab, Biobizkaia Health Research Institute, Barakaldo, Spain; 11 IKERBASQUE Basque Foundation for Science, Bilbao, Spain; 12 Center for Inflammation Imaging, Department of Radiology, Massachusetts General Hospital, Harvard Medical School, Boston, Massachusetts, USA; 13 Boston University, Chobanian & Avedisian School of Medicine, Boston, MA, USA; 14 Department of Genetics and Genomics Sciences, Ronald M. Loeb Center for Alzheimer’s Disease, Nash Family Department of Neuroscience, Icahn School of Medicine at Mount Sinai, New York, NY, USA; 15 UK Dementia Research Institute, University College London, London, UK

**Keywords:** Alzheimer's disease, AD, human astrocytes, hAstrocytes, human induced pluripotent stem cells, hiPSCs, chimeric mice, apolipoprotein E, APOE, amyloid-beta, Aβ

## Abstract

Astrocytes and APOE are strongly implicated in Alzheimer’s disease (AD), yet the impact of astrocytes carrying different *APOE* variants on AD hallmarks remains incompletely understood. Here, we generate a chimeric model of AD by transplanting isogenic *APOE3* or *APOE4* human induced pluripotent stem cell-derived astrocyte progenitors into neonatal AD mice. Donor cells differentiate into human astrocytes that integrate into the cortex and display morphologies consistent with interlaminar-like astrocytes. *APOE3* and *APOE4* astrocytes differ in expression of APOE, which associates differentially with Aβ plaques. Notably, *APOE3* astrocytes are associated with reduced Aβ burden, Tau pathology, and neuritic dystrophy, whereas *APOE4* astrocytes exacerbate these processes. They also induce distinct microglial responses: *APOE4* astrocytes enhance microglial clustering around Aβ plaques and promote a disease-associated microglia-like state, whereas *APOE3* astrocytes reduce clustering and support a more homeostatic profile. These findings highlight a role for human astrocytes and *APOE*-dependent astrocyte functions in modulating AD-related pathology.

## Highlights

• *APOE3* and *APOE4* hiPSC-derived astrocyte progenitors engraft the mouse brain
• *APOE3* and *APOE4* hAstrocytes exhibit distinct APOE expression and plaque associations
• *APOE3* hAstrocytes attenuate Aβ burden, Tau pathology and neuritic dystrophy
• *APOE4* hAstrocytes promote microglial clustering and DAM-like states

## Graphical abstract

**Figure:**
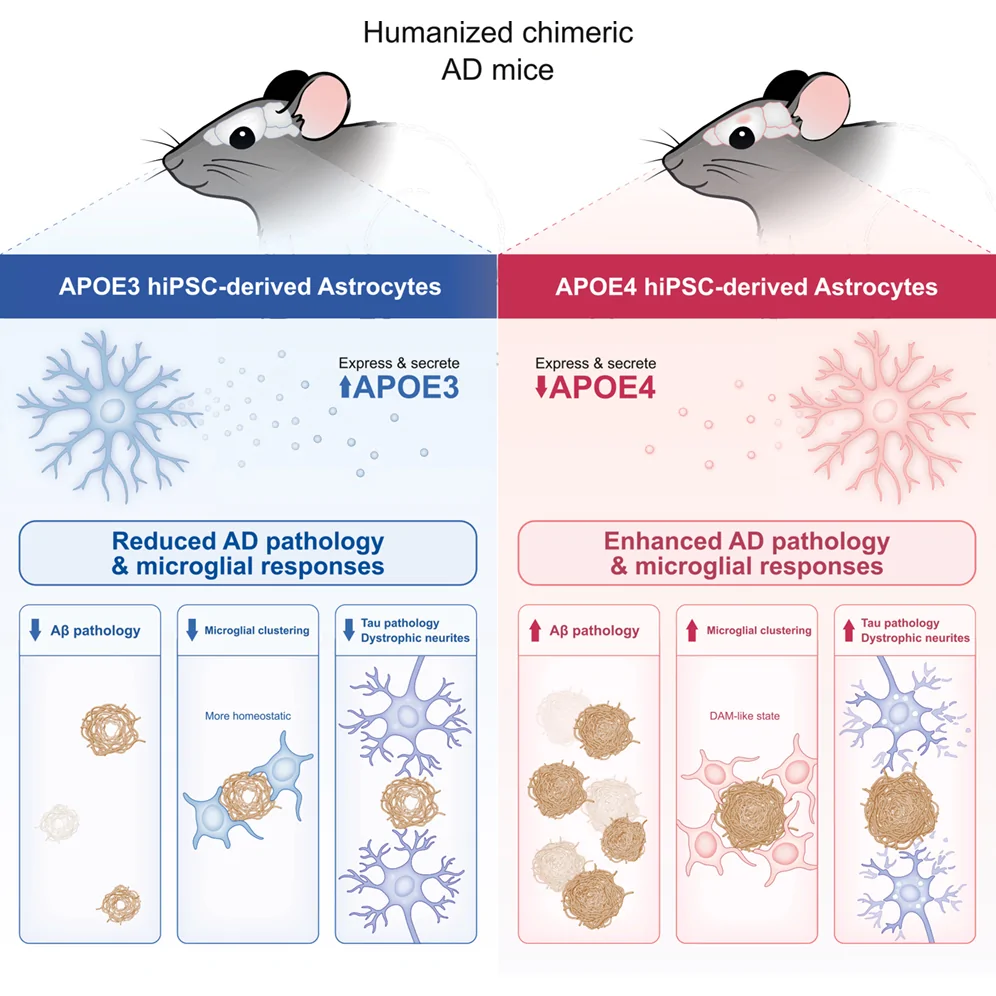

## Teaser

Cruz-Sese et al. generate chimeric Alzheimer’s disease mice with *APOE3* or *APOE4* hiPSC-derived astrocytes. *APOE3* hAstrocytes reduce amyloid burden, Tau pathology and neuritic dystrophy, while *APOE4* hAstrocytes exacerbate these processes. Both variants distinctly shape microglial states and responses, revealing an APOE-dependent role for human astrocytes in modulating Alzheimer’s disease pathology.

## Introduction

The apolipoprotein E (*APOE4*) variant of the *APOE* gene is the strongest genetic risk factor for late-onset Alzheimer’s disease (LOAD). *APOE* encodes three common alleles (*E2*, *E3*, *E4*) associated with differential AD risk.^1^ *APOE3* is the most frequent and considered the neutral allele; *APOE4* significantly increases AD risk in a gene dose-dependent manner, and *APOE2* is associated with reduced risk and later age at onset.^2^ Within the brain, APOE is predominantly expressed and produced by astrocytes under homeostatic conditions, whereas microglia and neurons upregulate APOE expression following injury.^3^^,^^4^ Although APOE's canonical function is to regulate cholesterol homeostasis through lipid efflux, it also plays essential roles for CNS health and contributes to AD progression.^1^^,^^2^ However, how distinct *APOE* variants influence AD pathogenesis and how they affect different brain cell types remain poorly defined.

Recent studies indicate that *APOE* isoforms shape astrocyte and microglial molecular states and functions in AD.^5^^,^^6^^,^^7^^,^^8^^,^^9^ In astrocytes specifically, *APOE* variants differentially affect AD-associated features such as Aβ burden, synapse loss, and Tau-mediated neurodegeneration.^10^^,^^11^^,^^12^^,^^13^^,^^14^^,^^15^^,^^16^ Most of this knowledge derives from studies in AD mouse models or human induced pluripotent stem cell (hiPSC)-derived *in vitro* systems. While informative, key species-specific differences exist between rodent and human astrocytes. Human astrocytes are 2–3 times larger and more complex than rodent astrocytes, have more processes, and contact more synapses.^17^^,^^18^^,^^19^ Besides, human and mouse astrocytes display distinct gene expression profiles,^19^ and importantly, the *APOE* polymorphism is absent in rodents. Functionally, human astrocytes display different responses when exposed to inflammatory stimuli^20^^,^^21^^,^^22^ and propagate calcium waves faster than rodent ones.^18^^,^^19^^,^^23^ Moreover, AD mouse models are unable to recapitulate some key human-specific cellular hallmarks of LOAD.^24^ Due to these critical differences, and in order to study human-specific aspects of AD, many groups are using stem cell technologies and hiPSCs from patients with AD and healthy individuals. The ability to generate hiPSC-derived astrocytes^25^^,^^26^^,^^27^ and other CNS cell types is offering new opportunities to model AD *in vitro*.^28^ Yet, hiPSC-derived neurons and glia grown in culture show limited maturation and lack essential components present in the brain, often resulting in phenotypes and transcriptional states that diverge from those in the human brain.^19^^,^^25^^,^^29^

To address these challenges and to examine how *APOE3* and *APOE4* human astrocytes influence major AD hallmarks in the living brain, we used a xenotransplantation model in which hiPSC-derived astrocyte progenitors were engrafted into the brains of AD model mice. In this chimeric model, *APOE3* and *APOE4* progenitors differentiated similarly into mature astrocytes (hAstrocytes) that integrated predominantly into upper layers of one cortical hemisphere and acquired morphological features consistent with interlaminar astrocytes, a population largely restricted to humans and hominids.

Strikingly, in AD chimeric mice, *APOE3* and *APOE4* hAstrocytes differed markedly in their APOE expression patterns and exerted opposing effects on AD-related processes. Cortical regions hosting *APOE4* hAstrocytes exhibited increased Aβ burden, Tau pathology, neuritic dystrophy, and plaque-associated microglia responses. In contrast, cortical regions with isogenic *APOE3* hAstrocytes showed reductions in these pathological hallmarks. Together, our *in vivo* findings demonstrate that hAstrocytes carrying *APOE3* or *APOE4* variants and the distinct APOE isoforms they produce can ameliorate or exacerbate not only Aβ pathology, but also Tau pathology, neuritic dystrophy, and microglial activation. These results highlight a more substantial contribution of hAstrocytes to AD progression and neurodegeneration than previously appreciated and validate chimeric mouse models as a powerful platform for dissecting human astrocyte biology and their contribution to AD.

## Results

### Transplanted hiPSC-derived astrocyte progenitors integrate efficiently within the mouse host brain and differentiate to human astrocytes

**Figure 1:**
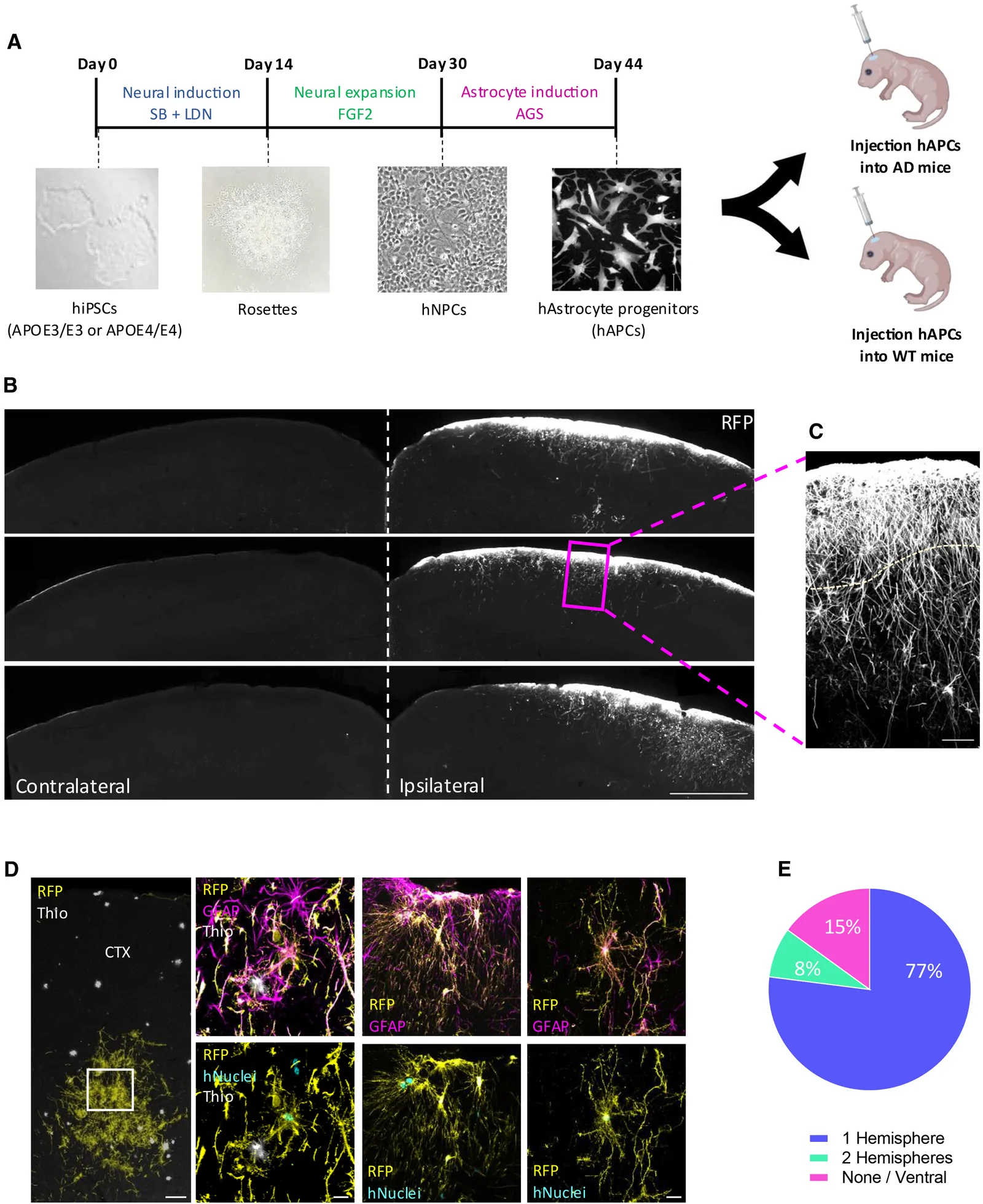
Transplanted cells integrate efficiently within the mouse host brain and differentiate to human astrocytes (A) Schematics of the differentiation and transplantation procedures. hiPSCs: human induced pluripotent stem cells, hNPCs: human neural progenitor cells, hAPCs: human astrocyte progenitor cells, SB: SB431542, LDN: LDN193189, FGF2: fibroblast growth factor 2, AGS: astrocyte growth supplement. (B) Overview of the chimeric cortex showing interlaminar astrocytes (RFP, white) in Layer I of one cortical hemisphere of chimeric brains 5 months after transplantation. Scale bars, 500 μm. (C) High-magnification view of hAstrocytes in mouse cortex layer I. Scale bars, 50 μm. (D) Overview and higher magnification images of hAstrocytes (RFP, yellow; GFAP, magenta or hNuclei, cyan) integrated throughout the forebrain. Aβ plaques are stained with Thioflavin S (Thio, white). Scale bars, 20 μm in high-magnification images and 100 μm in overview. (E) Pie chart shows the percentage of chimeric brains with cortical integration of hAstrocytes in one hemisphere, in the two hemispheres, or no cortical integration (*n* = 53 mice; *APP PS1 Prkdc*^scid/scid^).

To generate chimeric mice with human astrocytes, we employed a similar approach to the one we used before.^8^ Briefly, we differentiated eight hiPSC lines from three patients with AD carrying either the *APOE4/E4 (APOE4)* alleles or the corresponding corrected *APOE3/E3 (APOE3)* isogenic lines (Table S1) into astrocyte progenitor cells (hAPCs) *in vitro* following an established protocol^26^ (Figure 1A). hAPCs showed comparable morphology across lines and similarly expressed astrocyte markers (Figures S1A and S1B). After 44 days in culture, we transplanted the tdTomato-expressing hAPCs into the brains of newborn wildtype and AD mice (Figure 1A). Two mouse models suitable for transplantations were used: *APP PS1 Prkdc*^scid/scid^ mice and *App*^*NL-G-F*^ *Rag2*^−/−^ mice. Chimeric mice were aged for five to six months, when main AD pathological hallmarks are well established in these AD model mice, and their brains were collected for immunofluorescence (IF), MSD-ELISA, and bulk RNA-seq analyses.

Transplanted cells were identified by IF analyses based on the expression of the tdTomato marker RFP and the human nuclear antigen hNuclei (Figure S1C). These cells efficiently integrated into the mouse host brain and established contacts with blood vessels (Figure S1D), consistent with our previous findings.^8^ Both *APOE3* and *APOE4* hAPCs exhibited comparable engraftment patterns, with transplanted cells predominantly infiltrating the forebrain. In nearly 80% of chimeric mice, a large proportion of the transplanted cells integrated into the upper layers of one cortical hemisphere (Figures 1B, 1C, and 1E) and displayed astrocytic identity: they expressed canonical astrocyte markers including GFAP, vimentin, aquaporin, and S100B (Figure S1E) and lacked expression of neuronal or oligodendrocyte markers (Figure S1F). Notably, these transplanted cells located in Layer 1 of the human cortex exhibited morphological features characteristic of interlaminar astrocytes,^30^^,^^31^ including small, round cell bodies near the pial surface (Figure S1C) and long, unbranched processes extending into deeper cortical layers (Figures 1B and 1C), consistent with our earlier findings.^8^ This morphology is not observed in the same human astrocytes maintained *in vitro* (Figure S1A). In addition, a small subset of transplanted cells integrated individually throughout the forebrain, adopting other morphologies (Figure 1D). Engraftment efficiency varied across hiPSC lines but was independent of patient background or *APOE* genotype (Table S2). For downstream analyses, we selected four of the eight hiPSC lines that showed the highest engraftment efficiency (lines 5–8 in Table S2). In addition, given that the most consistent and abundant population of engrafted cells was interlaminar-like astrocytes within the upper layers of one cortical hemisphere (Figures 1B, 1C, and 1E), we focused our analyses on this region and astrocyte subtype. To ensure rigor and consistency, we only included mice showing comparable patterns of integration in the analyses. Comprehensive details regarding the specific isogenic pairs and number of mice used in each analysis are provided in Table S3.

Together, these data demonstrate robust engraftment and maturation of transplanted hAPCs into human astrocytes (hAstrocytes) in both wild-type and AD mouse cortex. By establishing a chimeric mouse system with hAstrocytes, our work provides a powerful, unique platform to investigate their contribution to AD pathophysiology *in vivo*.

### hAstrocytes express APOE in an isoform-dependent manner

**Figure 2:**
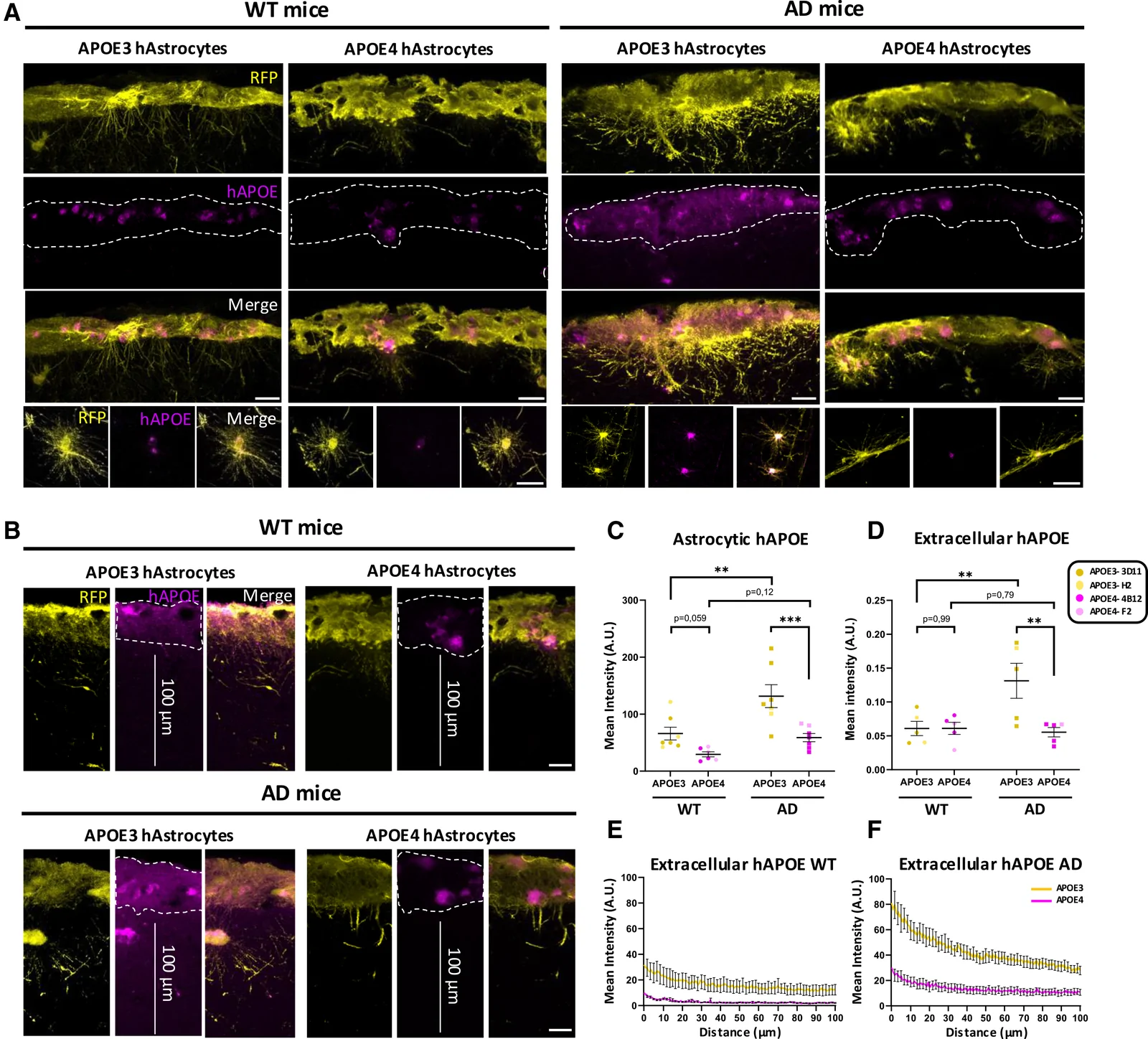
hAstrocytes express APOE in an isoform-dependent manner (A, B) Representative images of the chimeric cortex showing *APOE3* and *APOE4* hAstrocytes (RFP, yellow) and human APOE (hAPOE, magenta) in 5–6 months old WT and AD mice. Top and bottom images represent separate fields. Scale bars, 20 μm in top images and 200 μm in bottom images. (C) Quantification of the hAPOE intensity per area in *APOE3* and *APOE4* hAstrocytes in chimeric mice (*APP PS1 Prkdc*^scid/scid^; *n* = 7 WT mice with *APOE3* and *n* = 6 WT mice with *APOE4* hAstrocytes; *n* = 7 AD mice with *APOE3* and *n* = 7 AD mice with *APOE4* hAstrocytes). (D) Quantification of the mean intensity of extracellular hAPOE in WT and AD mice hosting *APOE3* and *APOE4* hAstrocytes (*APP PS1 Prkdc*^scid/scid^; *n* = 5 WT mice with *APOE3* and *n* = 5 WT mice with *APOE4* hAstrocytes; *n* = 5 AD mice with *APOE3* and *n* = 5 AD mice with *APOE4* hAstrocytes). Data are represented as mean ± SEM, two-way ANOVA with Holm-Sidak test; ns = not significant, ^∗^*p* < 0.05, ^∗∗^*p* < 0.01, and ^∗∗∗^*p* < 0.001. (E, F) Mean intensity of extracellular hAPOE along a distance of 100 μm from the soma of hAstrocytes in both WT and AD mice.

APOE is primarily expressed and produced by astrocytes in homeostatic conditions, whereas both microglia and neurons also upregulate APOE under pathological conditions.^3^^,^^4^ We therefore asked whether hAstrocytes expressed human APOE (hAPOE) *in vivo* within the chimeric mouse brain, and whether this expression differed between isoforms. To address this, we analyzed the expression of hAPOE in transplanted hAstrocytes by IF using a monoclonal antibody specific for human APOE. We observed higher hAPOE expression in *APOE3* hAstrocytes compared with *APOE4* hAstrocytes in both wildtype and AD mice (Figures 2A and 2C), consistent with previous findings in cultured astrocytes.^13^^,^^14^ Moreover, we observed that while *APOE3* hAstrocytes exhibited a significant upregulation of hAPOE in AD mice relative to wildtype controls, *APOE4* hAstrocytes showed no significant change under the same conditions (Figures 2A and 2C).

Since APOE is secreted to the extracellular space, we next examined whether there were isoform-dependent differences in extracellular hAPOE. Quantification of extracellular hAPOE by IF within 100 μm of the hAstrocyte transplants revealed higher levels in regions surrounding *APOE3* hAstrocytes than those near *APOE4* hAstrocytes in AD mice (Figures 2B and 2D). Furthermore, extracellular hAPOE was significantly elevated around *APOE3* hAstrocytes in AD mice compared with wildtype mice, whereas levels remained unchanged near *APOE4* hAstrocytes (Figures 2B–2F). To validate these findings, we quantified hAPOE protein levels in cortical homogenates from chimeric brains using a human APOE-specific ELISA. Both soluble and insoluble hAPOE levels were significantly higher in samples containing *APOE3* hAstrocytes compared with those hosting *APOE4* hAstrocytes (Figures S2A and S2B).

Together, these results demonstrate isoform-dependent regulation of hAPOE expression by hAstrocytes *in vivo*, under both control and AD conditions, with reduced intracellular and extracellular hAPOE in *APOE4* hAstrocytes relative to their isogenic *APOE3* counterparts.

### APOE from hAstrocytes exhibits isoform-dependent binding to Aβ plaques

**Figure 3:**
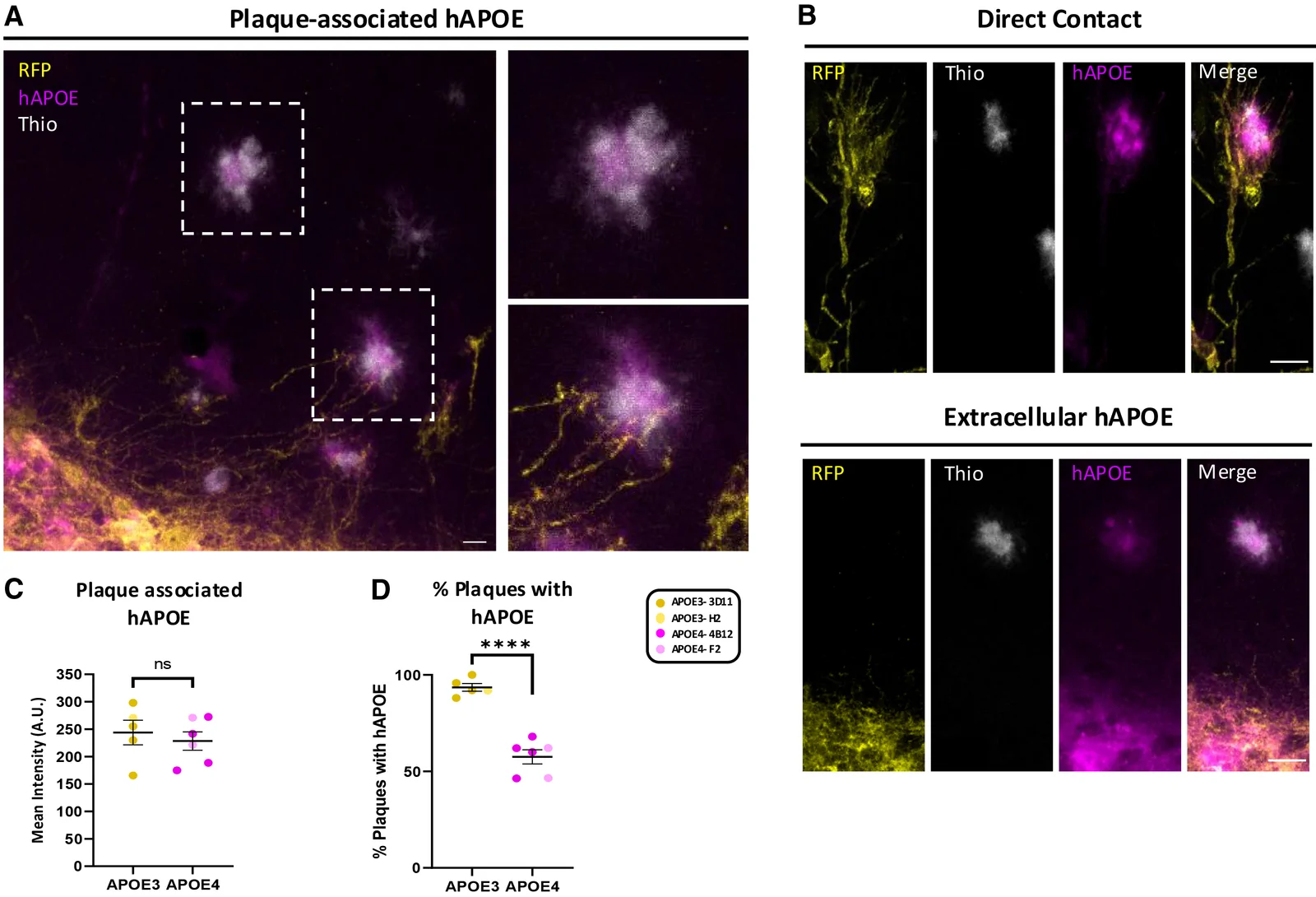
APOE from hAstrocytes binds to Aβ plaques (A) Representative images of hAstrocytes and their processes (RFP, yellow) and human APOE (hAPOE, magenta) that binds to Aβ plaques (Thio, white). Scale bars, 20 μm. (B) Both hAPOE from astrocyte processes in direct contact with the plaques (top image) and extracellular hAPOE (bottom image) bind to Aβ plaques. Scale bars, 20 μm. (C) Quantification of plaque-associated hAPOE mean intensity in AD chimeras with *APOE3* and *APOE4* hAstrocytes (*APP PS1 Prkdc*^scid/scid^; *n* = 5 AD mice with *APOE3* and *n* = 6 AD mice with *APOE4* hAstrocytes). (D) Percentage of plaques with hAPOE (*APP PS1 Prkdc*^scid/scid^; *n* = 5 AD mice with *APOE3* and *n* = 6 AD mice with *APOE4* hAstrocytes). Data are represented as mean ± SEM, unpaired *t* test; ns = not significant, ^∗^*p* < 0.05, ^∗∗^*p* < 0.01, and ^∗∗∗^*p* < 0.001.

Interestingly, hAPOE colocalized with amyloid-beta (Aβ) plaques in AD chimeric mice. We observed hAPOE at plaques in regions adjacent to hAstrocytes (Figure 3A), both at Aβ plaques in direct contact with astrocyte processes and at plaques not directly contacted by hAstrocytes (Figures 3A and 3B). This indicates that hAPOE in the extracellular space, as well as hAPOE from astrocyte processes contacting the plaques, can bind to Aβ deposits (Figure 3B). Quantification of hAPOE within plaques located within 100 μm of hAstrocyte transplants revealed comparable hAPOE levels in plaques near *APOE3* and *APOE4* hAstrocytes (Figure 3C). However, the percentage of plaques with hAPOE was significantly higher in regions next to *APOE3* hAstrocytes than close to *APOE4* hAstrocytes (Figure 3D). In contrast, hAPOE was not detected at plaques distant from hAstrocytes or in the contralateral hemisphere lacking hAstrocytes (Figure S2C).

Taken together with our earlier observations that *APOE3* hAstrocytes express higher levels of hAPOE than *APOE4* hAstrocytes, these findings suggest isoform-dependent differences in plaque engagement that may be influenced by hAPOE expression levels.

### APOE3 and APOE4 hAstrocytes differentially affect Aβ pathology

The integration pattern of hAstrocytes, predominantly occupying the upper layers of a single cortical hemisphere (Figures 1B and 1E), enabled us to assess their contribution to key AD pathological hallmarks within the same animal and brain section. To minimize inter-animal variability, we primarily compared the xenografted ipsilateral hemisphere with the contralateral, non-engrafted hemisphere. This internal control provides a robust baseline for detecting hAstrocyte-driven effects, as regional and animal-to-animal differences in pathology could otherwise confound interpretation. Importantly, the ipsilateral hemisphere reflects the combined effects of mouse and human APOE, whereas the contralateral hemisphere reflects mouse APOE alone. Direct comparisons of the effects of *APOE3* vs. *APOE4* hAstrocytes on AD pathological features are also provided.

**Figure 4:**
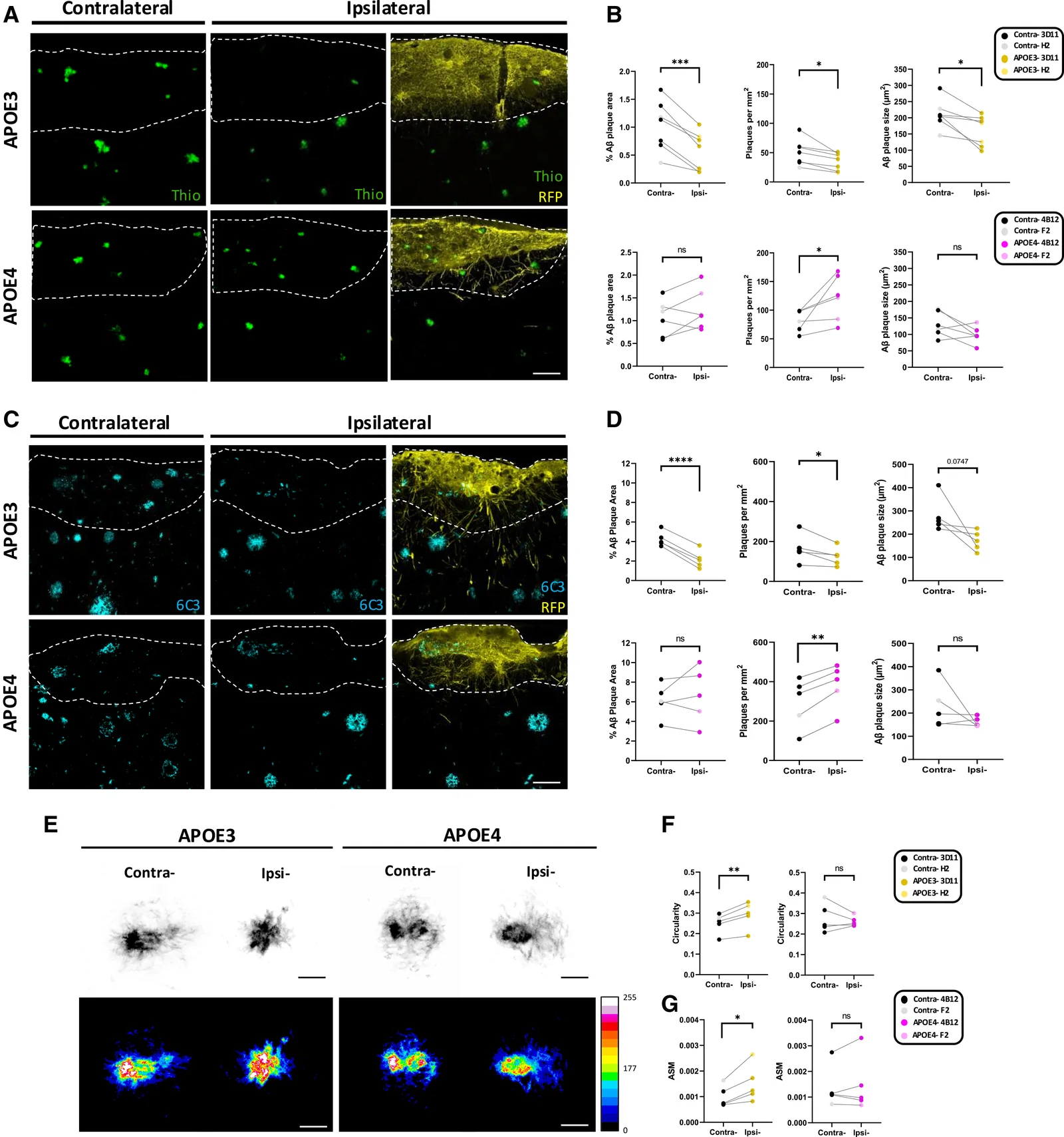
*APOE3* and *APOE4* hAstrocytes differentially affect Aβ pathology (A) Representative images of the cortex of AD chimeric mice 5–6 months after transplantation stained for hAstrocytes (RFP, yellow) and fibrillar Aβ (Thio, green). Both contralateral and ipsilateral cortices are shown. Scale bars, 50 μm. (B) Quantification of Aβ burden, number of plaques per area, and plaque size in cortex regions hosting *APOE3* or *APOE4* hAstrocytes and in contralateral cortex (*APP PS1 Prkdc*^scid/scid^; *n* = 7 mice with *APOE3* and *n* = 6 mice with *APOE4* astrocytes). (C) Representative images of the cortex of AD chimeric mice stained for hAstrocytes (RFP, yellow) and total Aβ (6C3, cyan). Scale bars, 50 μm. (D) Quantification of Aβ burden, number of plaques per area, and plaque size in cortex regions with *APOE3* or *APOE4* hAstrocytes and in contralateral areas (*APP PS1 Prkdc*^scid/scid^; *n* = 5 mice with *APOE3* and *n* = 5 mice with *APOE4* hAstrocytes). Data are represented as mean ± SEM, paired *t* test. (E) Representative images of *X*34-positive Aβ plaque conformation in grayscale and heat-density map based on intensity (red represents the highest intensity and blue is the lowest intensity) in contra- and ipsilateral cortices with *APOE3* and *APOE4* hAstrocytes. Scale bars, 10 μm. (F, G) Quantification of circularity (F) and ASM degree of compactness (G) of *X*34-positive plaques in contra- and ipsilateral cortices (*APP PS1 Prkdc*^scid/scid^; *n* = 5 mice with *APOE3* and *n* = 5 mice with *APOE4* hAstrocytes). Data are represented as mean ± SEM, paired *t* test; ns = not significant, ^∗^ = *p* < 0.05, ^∗∗^ = *p* < 0.01, and ^∗∗∗^ = *p* < 0.001.

We previously reported that transplanted hAstrocytes closely interact with Aβ plaques in AD mouse brains.^8^ This provides a unique opportunity to examine whether hAstrocytes carrying different *APOE* isoforms differentially influence Aβ pathology. Using Thioflavin S to label dense-core fibrillar plaques in cortical sections of 5–6 months-old chimeric AD mice, we found a significant reduction in Aβ load in cortical regions hosting *APOE3* hAstrocytes compared with contralateral regions (Figures 4A and 4B). In contrast, regions containing *APOE4* hAstrocytes showed an increased number of plaques per area but no significant changes in plaque size or total area covered by plaques (Figures 4A and 4B). To assess overall Aβ deposition, we performed staining with the Aβ-specific monoclonal antibody 6C3. 6C3 staining yielded consistent results: total amyloid burden decreased in cortical regions hosting *APOE3* hAstrocytes compared with contralateral regions (Figures 4C and 4D), while there were more Aβ plaques per area in regions where *APOE4* hAstrocytes were present compared with their contralateral sides (Figures 4C and 4D). Similar trends were observed when quantifying Aβ load in cortical areas within 100 μm of the transplanted cells (Figures S3A and S3B). These findings suggest that both hAstrocytes and the hAPOE they produce influence Aβ deposition *in vivo*. To further examine Aβ accumulation, we quantified soluble and insoluble Aβ_42_ levels by ELISA. In AD chimeras, both forms of Aβ_42_ were significantly reduced in regions hosting *APOE3* hAstrocytes compared with contralateral areas (Figure S3C). In contrast, no significant differences were observed in the presence of *APOE4* hAstrocytes (Figure S3C). We also measured both soluble and insoluble Aβ_42_ levels in wildtype chimeras and found minimal levels of Aβ_42_ (Figure S3D), indicating that hAstrocytes do not produce Aβ in this model at this stage. Collectively, these results indicate that both *APOE3* and *APOE4* hAstrocytes and the hAPOE they produce modulate Aβ deposition into fibrillar Aβ plaques.

We next investigated plaque morphology by quantifying circularity and compactness of X-34-stained Aβ plaques. Plaques associated with *APOE3* hAstrocytes were more circular and compact compared with plaques in contralateral regions (Figures 4E–4G), whereas plaques near *APOE4* hAstrocytes showed no significant differences in these parameters (Figures 4E–4G).

Together, these findings demonstrate a critical role for hAstrocytes and hAPOE in shaping Aβ pathology *in vivo* in chimeric AD mice, and reveal clear isoform-specific effects. *APOE3* hAstrocytes and hAPOE3 reduce plaque burden and promote plaque compactness, whereas *APOE4* hAstrocytes and hAPOE4 exert a more modest effect, increasing plaque number without altering plaque size or morphology. Direct comparison of *APOE3* vs. *APOE4* hAstrocytes across all these features (Figure S4) further reinforces their opposing contributions to overall Aβ pathology.

### Human brains from patients with AD harboring APOE4 alleles exhibit increased Aβ load

To assess the translational relevance of our findings, we analyzed Aβ load and hAPOE expression at plaques in the lateral temporal cortex of patients with AD carrying either *APOE3/E3 (APOE3)* or *APOE4/E4 (APOE4)* alleles, all classified as Braak stages V–VI (Table S4). Thioflavin S staining revealed that patients with *APOE3* exhibited lower Aβ load in these cortical regions compared with patients with *APOE4* (Figures S5A and S5B), consistent with previous reports^32^ and with our observations in chimeric mice (Figures 4A and 4B). We next examined hAPOE expression and found that hAPOE was detected at Aβ plaques in patients (Figure S5A), paralleling our chimeric mouse data. Interestingly, absolute hAPOE levels at the plaques were higher in patients with *APOE4* than in patients with *APOE3* (Figure S5C), in contrast with our chimeric mouse findings, where plaque-associated hAPOE levels were comparable between isoforms (Figure 3C). This discrepancy likely reflects the fact that, in human brains, hAPOE is produced not only by astrocytes but also by microglia and neurons, which upregulate APOE expression under pathological conditions.^4^

### APOE3 and APOE4 hAstrocytes distinctly shape microglial states and responses to Aβ plaques

**Figure 5:**
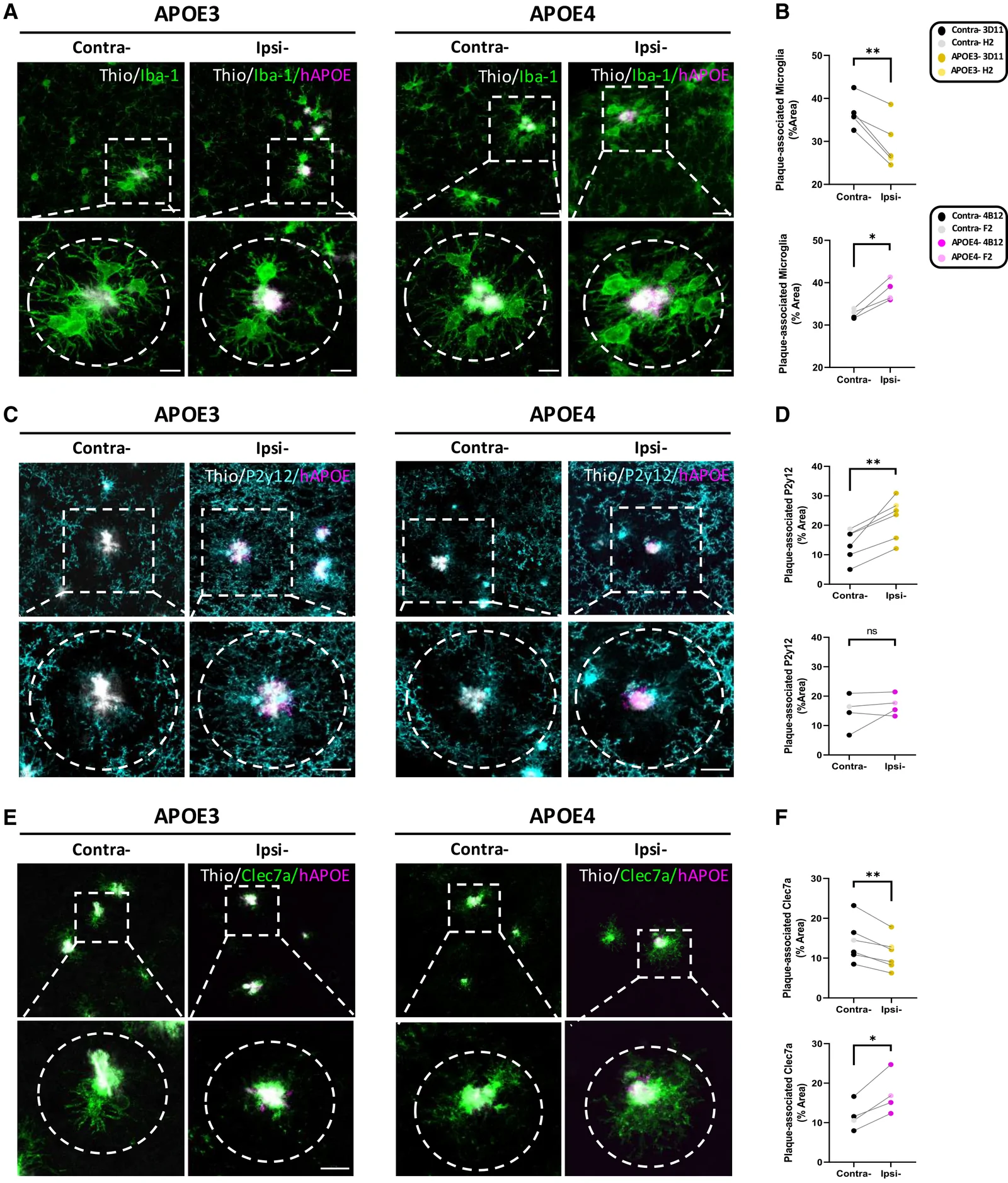
*APOE3* and *APOE4* hAstrocytes and hAPOE differentially modulate microglial responses (A) Representative images of Iba-1 (green) positive microglia around Aβ plaques (Thio, white) with or without human APOE (hAPOE, magenta) in contralateral and ipsilateral cortices of AD chimeras hosting *APOE3* and *APOE4* hAstrocytes 5–6 months after transplantation. Scale bars, 10 μm. (B) Percentage area covered by Iba-1 positive microglia around Aβ plaques in AD chimeras (*APP PS1 Prkdc*^scid/scid^; *n* = 5 mice with *APOE3* and *n* = 4 mice with *APOE4* hAstrocytes). (C) Representative images of P2y12 (cyan) positive microglial cells surrounding Aβ plaques (Thio, white) with and without human APOE (hAPOE, magenta) in contra- and ipsilateral cortices of AD chimeras. Scale bars, 10 μm. (D) Percentage area covered by P2y12 positive microglia around plaques (*APP PS1 Prkdc*^scid/scid^; *n* = 6 mice with *APOE3* and *n* = 4 mice with *APOE4* hAstrocytes). (E) Representative images of Clec7a (green) positive microglial cells surrounding Aβ plaques (Thio, white) with and without human APOE (hAPOE, magenta) in contra- and ipsilateral cortices of AD chimeras. Scale bars, 10 μm. (F) Percentage area covered by Clec7a positive microglia around plaques (*APP PS1 Prkdc*^scid/scid^; *n* = 6 mice with *APOE3* and *n* = 4 mice with *APOE4* hAstrocytes). Data are represented as mean ± SEM, paired *t* test; ns = not significant, ^∗^ = *p* < 0.05, ^∗∗^ = *p* < 0.001, and ^∗∗∗^ = *p* < 0.0001.

Aβ pathology is characterized by the clustering of microglia around amyloid plaques. Because microglial responses are known to be modulated by *APOE* isoforms,^12^^,^^32^^,^^33^^,^^34^ we next investigated whether *APOE3* and *APOE4* hAstrocytes and hAPOE isoforms differentially affect the responses of microglia to amyloid plaques in our chimeric mice. We performed IF using Iba-1, a myeloid cell marker upregulated in microglia, together with Thioflavin S to label Aβ plaques, and an antibody that specifically detects hAPOE. Microglial clustering was quantified around plaques in cortical regions within 100 μm of hAstrocytes and compared with contralateral, non-engrafted hemispheres. To control for potential confounding effects of plaque size, only plaques of comparable sizes were analyzed (Figure S8B). Notably, in cortical regions near *APOE3* hAstrocytes, microglial clustering around hAPOE positive plaques was significantly reduced compared with contralateral regions (Figures 5A and 5B). In contrast, areas adjacent to *APOE4* hAstrocytes showed increased microglial clustering around hAPOE containing plaques relative to the contralateral side (Figures 5A and 5B). When examining total microglia, both plaque-associated and non-plaque-associated, we found that cortical regions containing *APOE3* hAstrocytes exhibited decreased total microglia area coverage, whereas regions with *APOE4* hAstrocytes showed no significant change compared with their respective contralateral hemispheres (Figures S6A and S6B). These observations prompted us to examine microglial activation around plaques. We first assessed expression of P2y12 and Clec7a, markers of homeostatic and disease-associated microglia (DAM), respectively. Plaque coverage by P2y12 positive microglia was significantly increased in areas near *APOE3* hAstrocytes but unchanged near *APOE4* hAstrocytes relative to their contralateral sides (Figures 5C and 5D). Conversely, Clec7a positive microglial coverage decreased near *APOE3* hAstrocytes, whereas it increased near *APOE4* hAstrocytes relative to their contralateral sides (Figures 5E and 5F). Direct comparison of *APOE3* vs. *APOE4* hAstrocytes (Figures S6C–S6I) further underscores the distinct effects of each variant shaping microglial responses.

**Figure 6:**
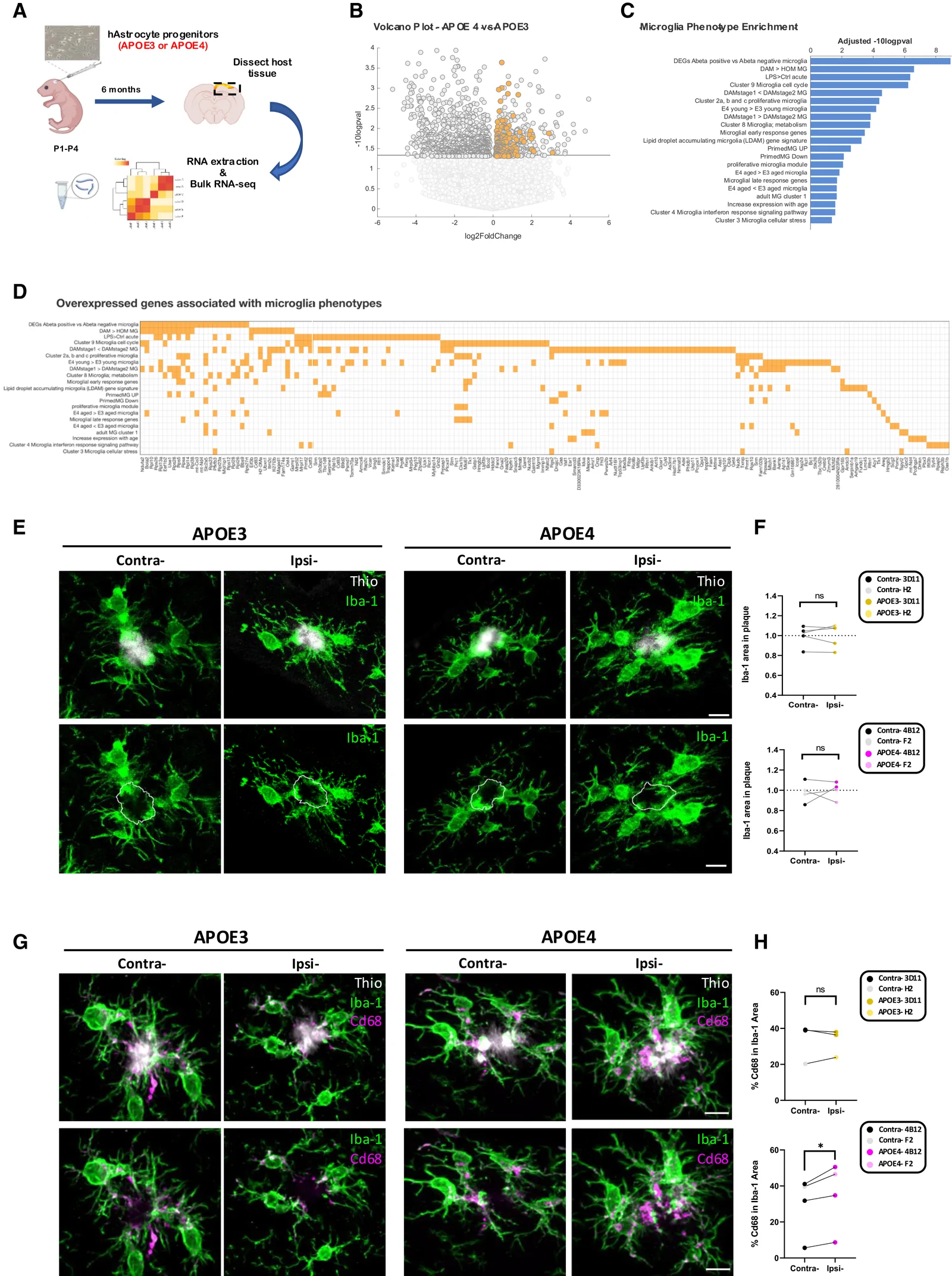
*APOE4*-driven transcriptional changes in host microglia (A) Experimental design for RNA sequencing experiments. P1-P4 mice (*App*^*NL-G-F*^*Rag2*^−/−^) were injected with either *APOE3* or *APOE4* hAstrocyte progenitor cells. Six months after transplantation, host tissue surrounding transplanted astrocytes was dissected to perform RNA extraction and bulk sequencing. (B) Volcano plot of differential gene expression. Mouse-specific transcripts were quantified in brain tissue from AD mice engrafted with *APOE4* hAstrocytes (*n* = 6) compared to AD mice engrafted with *APOE3* hAstrocytes (*n* = 6). Each point represents a mouse gene. The x axis displays the log_2(fold change) and the *y* axis shows the -log10(*p* value). Differentially expressed genes (DEGs) were defined as having an adjusted *p* < 0.05 (horizontal line). Upregulated genes that are also part of the significantly enriched microglial gene sets are highlighted in orange. (C) Microglia phenotype enrichment analysis. The set of upregulated mouse genes in the *APOE4* group was analyzed using the *MGEnrichment* tool against a curated database of microglial gene signatures. The bar plot displays the top enriched studies/phenotypes, ranked by adjusted -log10(*p* value). Highly significant associations were found for signatures related to amyloid plaque-positive microglia and the DAM phenotype. (D) Overexpressed genes associated with microglia phenotypes. Heatmap shows the robust overlap between the set of *APOE4* upregulated DEGs (columns) and the significantly enriched microglial gene sets (rows) identified in (C). Orange boxes indicate the presence of a shared gene between the experimental DEG set and the established reference gene set, visually confirming the strong transcriptional alignment with established pathological microglial states. (E) Representative images of microglia (Iba-1, green) coverage of Aβ plaques (Thio, white) in contralateral and ipsilateral cortices of AD chimeras 5–6 months after transplantation. The perimeter of the plaque has been outlined with a white line. Scale bars, 10 μm. (F) Percentage of Aβ plaque area occupied by Iba-1-positive microglia, normalized to the contralateral area near hAstrocytes in AD chimeras (*APP PS1 Prkdc*^scid/scid^; *n* = 5 mice with *APOE3* and *n* = 4 mice with *APOE4* hAstrocytes). (G) Representative images show microglia (Iba-1, green), Cd68 (magenta), and Aβ plaques (Thio, white). Scale bars, 10 μm. (H) Percentage of Cd68 area within Iba-1 area around Aβ plaques (*APP PS1 Prkdc*^scid/scid^; *n* = 3 mice with *APOE3* and *n* = 4 mice with *APOE4* hAstrocytes). Data are represented as mean ± SEM, paired *t* test; ns = not significant, ^∗^ = *p* < 0.05, ^∗∗^ = *p* < 0.001, and ^∗∗∗^ = *p* < 0.0001.

To further investigate how *APOE3* and *APOE4* hAstrocytes influence microglial states, we performed RNA sequencing of cortical regions adjacent to transplanted cells and analyzed the differential gene expression profile of mouse-specific transcripts (Figure 6A). Specifically, we compared the transcriptomes of AD chimeric mice engrafted with *APOE4* hAstrocytes vs. those engrafted with *APOE3* hAstrocytes. This analysis identified 1,473 differentially expressed genes (DEGs, *p* < 0.05), with 495 genes upregulated and 978 downregulated in the *APOE4* group (Figure 6B; Table S5). We next used the *MGEnrichment* tool^35^ to evaluate associations between our DEGs and curated reference gene sets related to microglial development, function, and activation. The analysis revealed that genes significantly upregulated in the *APOE4* group showed a significant overlap with gene signatures characteristic of a DAM state and microglial responses to inflammatory stimuli (Figure 6C; Table S5). Notably, the most enriched signatures corresponded to amyloid plaque-associated microglia and DAM profiles compared with homeostatic microglia in AD models.^36^^,^^37^ (Figure 6C; Table S5). We filtered the curated *MGEnrichment* studies to highlight those most relevant to AD, aging, microglial subpopulation profiling via single-cell RNA-seq, and acute inflammatory responses such as LPS exposure. The complete list of enriched phenotypes is provided in Table S5. The heatmap (Figure 6D) further illustrates the shared gene overlap between our upregulated DEGs and these microglial reference signatures, confirming that *APOE4*-driven gene expression changes are the major contributors to the enrichment signal, and demonstrating a significant transcriptional overlap with multiple DAM-related and inflammatory microglial signatures. Examples include *Itm2c* (also known as *Cst7*), a canonical DAM marker; *Ifi27l2b*, an interferon-response gene linked to inflammatory microglial states; and *Cd83*, associated with antigen presentation and immune activation^36^^,^^37^ (Figure 6D).

Next, to further assess potential differences in microglia responses surrounding the plaques, we analyzed microglial coverage of Aβ plaques and the expression of Cd68, a lysosomal protein highly expressed in activated microglia in AD mice. Consistent with prior results, regions near *APOE4* hAstrocytes contained more microglia surrounding plaques, whereas areas near *APOE3* hAstrocytes exhibited fewer (Figure 6E). However, overall microglial plaque coverage did not differ between groups (Figures 6E and 6F), suggesting that microglia in *APOE3* regions may cover plaques more efficiently, requiring fewer cells to achieve comparable coverage. In addition, we found significantly higher levels of Cd68 in plaque-associated microglia close to *APOE4* hAstrocytes compared to those on the contralateral sides (Figures 6G and 6H), whereas no differences in Cd68 expression were found in the *APOE3* group (Figures 6G and 6H).

Collectively, these findings reveal a dynamic crosstalk between hAstrocytes, astrocytic hAPOE, and host microglia at Aβ plaques. *APOE4* hAstrocytes and hAPOE4 promote increased microglial clustering and a shift toward a DAM-like inflammatory state, whereas *APOE3* hAstrocytes and hAPOE3 favor reduced clustering and a more homeostatic microglial phenotype around plaques.

### APOE3 hAstrocytes ameliorate, whereas APOE4 hAstrocytes exacerbate neuritic dystrophy and Tau pathology

**Figure 7:**
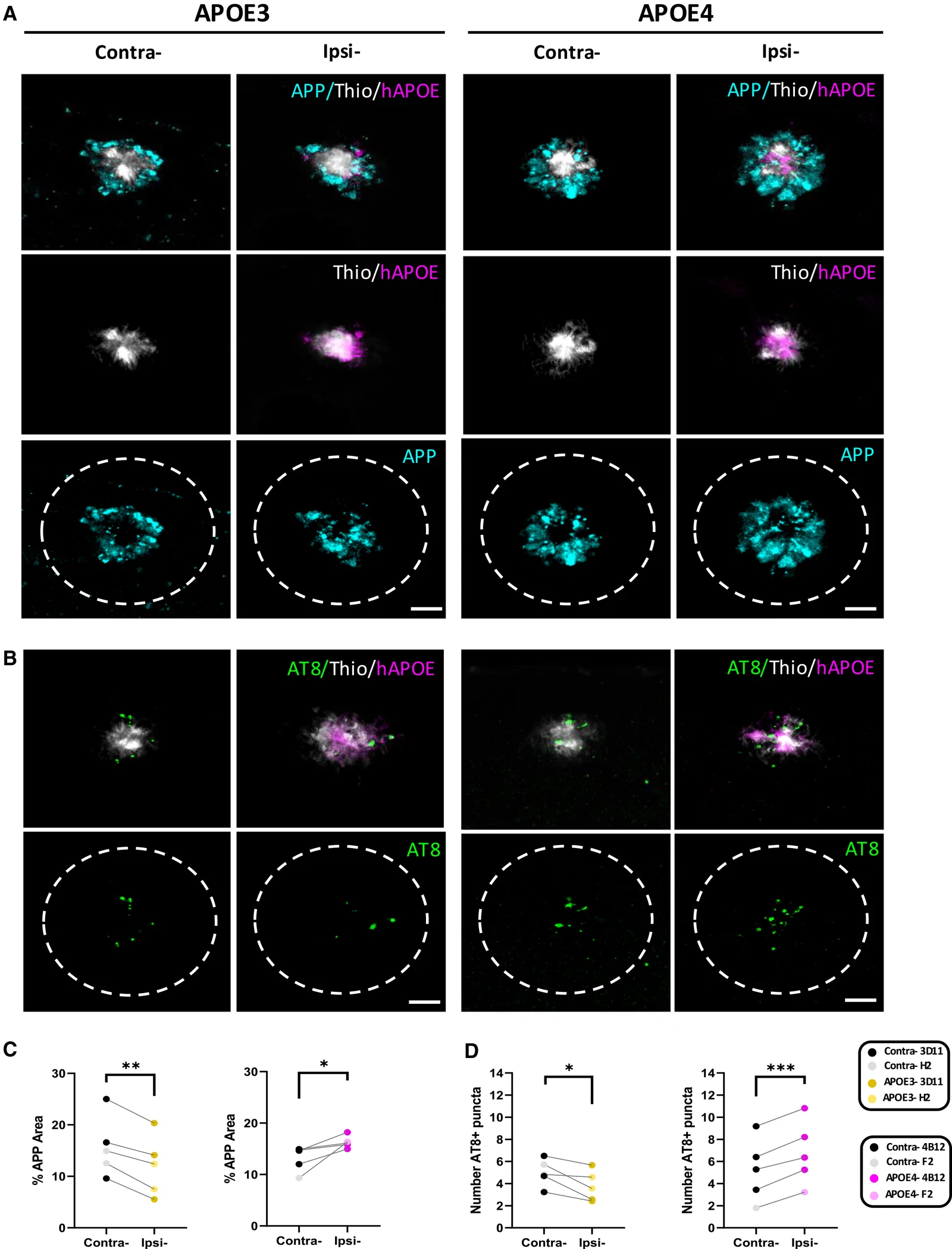
*APOE3* hAstrocytes ameliorate while *APOE4* hAstrocytes aggravate neuritic dystrophy and Tau pathology (A) Representative images of dystrophic neurites (APP, cyan) surrounding Aβ plaques (Thio, white) with and without human APOE (hAPOE, magenta) in contralateral and ipsilateral cortices of AD chimeras 5–6 months after transplantation. Scale bars, 10 μm. (B) Representative images of hyperphosphorylated Tau (AT8, green) surrounding Aβ plaques (Thio, white) with and without human APOE (hAPOE, magenta). Scale bars, 10 μm. (C) Percentage area occupied by APP positive dystrophic neurites around plaques in contra- and ipsilateral cortices (*APP PS1 Prkdc*^scid/scid^; *n* = 5 mice with *APOE3* and *n* = 5 mice with *APOE4* hAstrocytes). (D) Number of AT8 positive puncta around plaques in contra- and ipsilateral cortices (*APP PS1 Prkdc*^scid/scid^; *n* = 5 mice with *APOE3* and *n* = 5 mice with *APOE4* hAstrocytes). Data are represented as mean ± SEM, paired *t* test; ns = not significant, ^∗^ = *p* < 0.05, ^∗∗^ = *p* < 0.001, and ^∗∗∗^ = *p* < 0.0001.

Neuritic dystrophy and Tau hyperphosphorylation are key pathological features associated with Aβ plaques in both patients with AD and mouse models. We therefore investigated whether *APOE3* and *APOE4* hAstrocytes and hAPOE influence amyloid-associated neuronal damage and hyperphosphorylated Tau positive neuritic structures in chimeric AD mice. To assess neuritic dystrophy, we performed immunostaining for APP, which accumulates within dystrophic neurites, and quantified APP positive areas surrounding Aβ plaques of comparable size (Figure S8E). In cortical regions containing *APOE3* hAstrocytes, the area of APP positive dystrophic neurites was significantly reduced relative to contralateral, non-engrafted regions (Figures 7A and 7C; Figure S7A). Conversely, cortical areas containing *APOE4* hAstrocytes exhibited an increased area of APP positive dystrophic neurites compared with contralateral hemispheres (Figures 7A and 7C; Figure S7A). We next examined hyperphosphorylated Tau positive neuritic structures around Aβ plaques, hereafter referred to as Tau pathology, using the p-Tau specific antibody AT8. The number of AT8 positive puncta surrounding hAPOE positive plaques was markedly lower in cortical areas near *APOE3* hAstrocytes compared with contralateral regions (Figures 7B and 7D; Figures S7B and S7C). In contrast, plaques adjacent to *APOE4* hAstrocytes displayed an increased number of AT8 positive puncta relative to contralateral sides (Figures 7B and 7D; Figures S7B and S7C).

Overall, these results reveal that *APOE3* and *APOE4* hAstrocytes, and the hAPOE isoforms they produce, exert divergent effects on Tau pathology and neuritic dystrophy in chimeric mice. *APOE3* hAstrocytes and hAPOE3 attenuate both pathological features, whereas *APOE4* hAstrocytes and hAPOE4 exacerbate them, likely accelerating AD progression. Direct comparison of *APOE3* vs. *APOE4* hAstrocytes (Figures S7D–S7H) highlights the distinct contributions of each variant to these AD-related processes.

In summary, our findings underscore the contribution of hAstrocytes and astrocytic hAPOE to AD-associated hallmarks *in vivo*. *APOE3* hAstrocytes and hAPOE3 ameliorate Aβ pathology, Tau pathology, and neuritic dystrophy, and promote more homeostatic microglial responses (Table S6). In contrast, *APOE4* hAstrocytes and hAPOE4 aggravate these pathological features and drive an enhanced microglial clustering and DAM-like state (Table S6), thereby amplifying neuroinflammatory and degenerative cascades central to AD progression.

## Discussion

In this work, we expanded on previous efforts^8^ by generating chimeric mice transplanted with human isogenic astrocytes harboring either *APOE3* or *APOE4* alleles. We found that *APOE3* and *APOE4* astrocyte progenitors differentiated into mature astrocytes within the mouse brain and integrated predominantly into upper layers of the cortex. A substantial proportion of these astrocytes displayed morphological features characteristic of interlaminar astrocytes present in Layer 1 of the human cortex,^30^^,^^31^ consistent with our previous findings.^8^ Notably, this morphology is not observed in mouse astrocytes or in the same human astrocytes maintained *in vitro,* suggesting that human astrocytes preserve key human-specific structural traits within the mouse environment. Importantly, interlaminar astrocytes, largely unique to humans and hominids,^18^ have an elusive and poorly understood function, and their role in AD remains unexplored. Their relevance in AD may lie in their distinct cortical positioning and long-range processes, which may influence how astrocytes themselves and astrocytic APOE shape plaque-associated microenvironments. By establishing a chimeric mouse system harboring human astrocytes (hAstrocytes), our work provides a unique platform to investigate their contribution to AD pathophysiology *in vivo*.

Because astrocytes are major producers of APOE in the brain, we examined whether hAstrocytes expressed hAPOE *in vivo*, and whether *APOE3* and *APOE4* hAstrocytes differed in expression. Interestingly, *APOE4* hAstrocytes expressed lower levels of hAPOE than isogenic *APOE3* hAstrocytes in both wildtype and AD chimeric mice. Soluble and insoluble hAPOE levels were also reduced in regions containing *APOE4* hAstrocytes compared to *APOE3* hAstrocytes, indicating an isoform-dependent regulation of hAPOE expression in hAstrocytes *in vivo*. Consistently, studies in both human and mouse astrocytes in culture have reported similar reductions in APOE expression in *APOE4* relative to *APOE3* astrocytes, potentially due to post-translational modifications.^13^^,^^14^^,^^38^ Moreover, *APOE3* hAstrocytes upregulated hAPOE expression in AD compared with wildtype mice, whereas hAPOE levels remain unchanged in *APOE4* hAstrocytes, suggesting that such differences in hAPOE expression may influence AD progression. Remarkably, similar levels of hAPOE3 and hAPOE4 were detected within the plaques, but the proportion of plaques containing hAPOE was significantly higher in cortical areas close to *APOE3* hAstrocytes than in areas adjacent to *APOE4* hAstrocytes. These data reveal hAPOE isoform-dependent differences in plaque engagement that might be influenced by expression levels. We extended these analyses to human AD patient samples and observed APOE in Aβ plaques, consistent with our findings in chimeric mice. The presence of APOE in the plaques in both AD mice and patients with AD was also described in a recent study^39^ that suggests that APOE is indeed required for plaque formation.

We next investigated how *APOE3* and *APOE4* hAstrocytes and the hAPOE isoforms they produce influence **major AD pathological hallmarks**. Importantly, we found divergent effects of *APOE3* and *APOE4* hAstrocytes on **Aβ pathology**. Cortical regions hosting *APOE3* hAstrocytes showed reduced Aβ burden and lower soluble and insoluble Aβ_42_ levels. In contrast, regions containing *APOE4* hAstrocytes displayed more Aβ plaques per area compared with their respective contralateral sides. These findings suggest that *APOE4* hAstrocytes are less efficient than *APOE3* hAstrocytes at clearing Aβ *in vivo*. While these effects could be solely due to differences in astrocyte functionality in processing Aβ, similar effects were observed in adjacent cortical areas, suggesting that both hAstrocytes and the hAPOE they produce influence Aβ pathology *in vivo*. We could not detect soluble or insoluble Aβ_42_ levels in wildtype chimeras. These data indicate that transplanted hAstrocytes are not producing Aβ and contributing to plaque burden. Furthermore, plaques near *APOE3* hAstrocytes were more compact, whereas plaque compactness was unchanged near *APOE4* hAstrocytes, indicating that *APOE* variants also modulate plaque structure. Mirroring our findings in chimeras, analyses of patients with AD samples at Braak stages V–VI similarly revealed higher Aβ load in the lateral temporal cortex in *APOE4* compared with *APOE3* individuals, as described previously.^40^^,^^41^^,^^42^

We also uncovered opposing effects of *APOE3* and *APOE4* hAstrocytes on **Tau pathology and neuritic dystrophy.** Cortical regions hosting *APOE3* hAstrocytes showed fewer AT8 positive puncta and reduced area occupied by dystrophic neurites, whereas regions adjacent to *APOE4* hAstrocytes exhibited increased Tau pathology and neuritic dystrophy relative to their contralateral sides. Because these analyses were performed on plaques of similar size, the observed changes were not attributable to differences in plaque dimensions. These findings highlight the broader impact of hAstrocytes and hAPOE isoforms on AD progression, extending beyond Aβ to other key pathological processes.

A striking phenotype in AD chimeras was the differential response and activation state of **microglia surrounding Aβ plaques**. Regions containing *APOE3* hAstrocytes exhibited reduced microglial clustering and a more homeostatic state, marked by increased P2y12 and decreased Clec7a expression. In contrast, *APOE4* hAstrocytes promoted increased microglial clustering and elevated Clec7a expression. Consistent with our findings, patients with AD carrying *APOE4* show increased microglial clustering around plaques compared with non-carriers.^43^ These results support a model in which hAstrocytes engage in non-cell-autonomous crosstalk with host microglia, with astrocytic hAPOE contributing to signaling pathways that shape microglial responses and activation states.

Recent studies show a cell-autonomous role of *APOE4* microglia blocking the DAM response compared with *APOE3*.^6^^,^^33^^,^^34^ Intriguingly, *APOE4* hAstrocytes and hAPOE4 appear to exert opposite effects and bias microglia toward a dysfunctional state that interferes with proper responses to AD-associated pathology. Indeed, even though microglia increased clustering around plaques in *APOE4* chimeras, these cells fail to counteract Aβ pathology, Tau pathology, and neuritic dystrophy as efficiently as in *APOE3* chimeras. Interestingly, recent studies identified a reactive microglia population defined by specific expression of inflammatory signals whose frequency increased with *APOE4* burden. This population, called “terminally inflammatory microglia” (TIM), is functionally impaired, shows defects in Aβ clearance and an exhausted-like state, and has been found in both murine and human AD.^44^ While further profiling is required, our findings suggest that microglia in *APOE4* chimeras may adopt a similar impaired state. Transcriptomic profiling supports this interpretation. Bulk RNA-seq of cortical regions adjacent to *APOE3* or *APOE4* hAstrocytes identified nearly 1,500 differentially expressed mouse genes, with *APOE4* regions enriched for amyloid plaque-associated microglia, DAM, and inflammatory signatures.^36^^,^^37^ Upregulated genes included canonical DAM markers such as *Itm2c/Cst7*, interferon-responsive genes such as *Ifi27l2b*, and immune activation markers such as *Cd83*, indicating that *APOE4* hAstrocytes drive microglia toward a primed, inflammatory transcriptional state. In contrast, *APOE3* regions showed a more homeostatic profile. Furthermore, microglia located near *APOE4* hAstrocytes exhibited increased clustering and elevated Cd68 expression but did not effectively mitigate pathology, consistent with a maladaptive DAM-like or exhausted-like state. Conversely, microglia in *APOE3* regions displayed reduced activation and retained a more homeostatic phenotype. Together, these transcriptomic and histopathological data further indicate that *APOE3* and *APOE4* hAstrocytes differentially shape microglial responses and activation states, and contribute to their opposing effects on AD pathology.

Taken together, our findings provide compelling evidence for a critical role of hAstrocytes in AD progression and neurodegeneration. Specifically, these results reveal that *APOE3* and *APOE4* hAstrocytes shape multiple AD hallmarks and modulate host microglial responses, insights that were inaccessible through mouse-only or *in vitro* models. Our work also validates chimeric mice as a powerful platform for examining the contribution of hAstrocytes harboring different GWAS variants to AD and for dissecting their interactions with other CNS cell types.

### Limitations of the study

First, we used immune-deficient mice to enable the long-term survival of transplanted hAstrocytes. Although these mice lack adaptive immunity, their innate immune system remains largely intact, as shown previously^8^^,^^45^^,^^46^ and confirmed here. Moreover, transplanted hAstrocytes develop within the rodent brain, which is arguably a more physiological context for human cells than 2D and 3D culture systems, which also lack adaptive immunity. Two mouse models were used in this study. Importantly, the central conclusions of the study, that *APOE3* and *APOE4* hAstrocytes differentially modulate Aβ burden, Tau pathology, neuritic dystrophy and microglial responses, are derived from within-model comparisons, using ipsilateral versus contralateral hemispheres as internal controls. Thus, genotype-dependent effects are evaluated independently within each model, exploiting their specific strengths for distinct analyses. Besides, hAstrocytes derived from two pairs of isogenic hiPSC lines were used throughout the study, and in some analyses, an uneven number of replicates is shown for each line. While the number of replicates per line is not balanced, data from different patient lines follow a similar distribution and overall trend, and the consistency in behavior across lines supports our conclusions. In addition, differences between *APOE3* and *APOE4* groups are remarkable even though all analyses were performed in the presence of mouse endogenous APOE. Although remarkable, the observed effects are specific to plaque-associated pathology within the analyzed cortical regions and time window. Within these regions, all histological and APOE quantifications were restricted to areas with comparable graft size between conditions. Next, our analyses of patients with AD tissue were limited due to the small cohort of patient samples. While various studies in bigger cohorts of patients with AD samples support our findings, additional research will be needed to further confirm the phenotypes we described in this study. Finally, our work does not provide information about the molecular states of transplanted hAstrocytes within chimeric brains or full molecular dissection of the downstream mechanisms linking APOE genotype to pathological outcomes. Our goal was instead to address a fundamental and previously inaccessible question: how human astrocytes with distinct APOE genotypes influence AD-related pathology *in vivo*, within a physiological brain environment. The primary advance thus lies in establishing a human *in vivo* system that allows interrogating the contribution of hAstrocytes to AD pathology in a way that cannot be achieved using existing mouse or *in vitro* models. Single-cell or single-nucleus RNA sequencing and spatial transcriptomics will be essential to further delineate these cellular states and to define the mechanisms underlying pathological outcomes and astrocyte-microglia interactions *in vivo*. Such approaches may ultimately lead to therapeutic strategies aimed at shifting human astrocytes and microglia toward states that slow AD progression and neurodegeneration.

## Resource availability

### Lead contact

Requests for further information and resources should be directed to and will be fulfilled by the lead contact, Amaia M Arranz (amaia.arranz@achucarro.org).

### Materials availability

All unique/stable reagents generated in this study are available from the lead contact with a completed materials transfer agreement.

### Data and code availability

• Single-cell RNA-seq data have been deposited at GEO GSE335500 and are publicly available.
• Microscopy data reported in this paper will be shared by the lead contact upon request.
• This paper does not report original code.
• Any additional information required to reanalyze the data reported in this paper is available from the lead contact upon request.

## Acknowledgments

This project has been funded by the 10.13039/501100000780European Union (ERC, hASTROCURE, grant no. 101171591). Views and opinions expressed are, however, those of the authors only and do not necessarily reflect those of the European Union or the European Research Council. Neither the European Union nor the granting authority can be held responsible for them. This work was also funded by the 10.13039/501100004837Ministerio de Ciencia e Innovación: grant nos. PID2021-125443OB-100, PID2024-162968OB-I00 funded by MICIU/AEI/https://doi.org/10.13039/501100011033 and by 10.13039/501100008530ERDF, EU and grant no. RYC2020-029494-I funded by MICIU/AEI/https://doi.org/10.13039/501100011033 and ESF Investing in your future, and the 10.13039/100000957Alzheimer's Association (AARG-21-850389). hiPSC astrocyte progenitor derivation was supported by 10.13039/100000002NIH NIA R01AG082362, R01AG083941, and U19AG069701. Mouse experiments were supported by the animal facility of the University of the Basque Country (UPV/EHU). We want to particularly thank the patients and the Biobank Banco de Tejidos CIEN for their collaboration. We also thank the laboratory of Prof. David Holtzman for sharing protocols for IF, and Dr. Estibaliz Santiago-Mujika for revising the last version of the manuscript.

## Author contributions

Conceptualization, A.M.A.; methodology, A.M.A., B.D.S., and J.C.S.; investigation, J.C.S., M.M.A., M.A.T., J.O., L.R., N.G.G., L.C., L.E., P.P., A.S., A.A., and I.D.; writing – original draft, A.M.A. and J.C.S.; writing – review and editing, all authors; funding acquisition, A.M.A.; resources, T.S., T.C.S., L.R.A., A.R.G., J.TCW., and A.G.; supervision, A.M.A. and E.A.

## Declaration of interests

B.D.S. has been a consultant for Eli Lilly, Biogen, Janssen Pharmaceutica, Eisai, AbbVie, and other companies and is now a consultant to Muna Therapeutics. B.D.S is a scientific founder of Augustine Therapeutics and a scientific founder and stockholder of Muna Therapeutics. J.TCW. serves on the scientific advisory board of NeuCyte, Inc. and has consulted for CareCureSystems Corporation. The remaining authors declare no competing interests.

## Declaration of generative AI and AI-assisted technologies in the writing process

During the preparation of this work, the authors used ChatGPT in order to correct grammar. After using this tool, the authors reviewed and edited the content as needed and take full responsibility for the content of the publication.

## STAR★Methods

### Key resources table

**Table:** 

REAGENT or RESOURCE	SOURCE	IDENTIFIER
**Antibodies**		

4G8	BioLegend	Cat# 800703; RRID: AB_662812
Amyloid beta MOAB-2 6C3	Merck	Cat# MABN254; RRID: AB_2895168
APC	Millipore	Cat# OP80; RRID: AB_2057371
APP A4	Millipore	Cat# MAB348; RRID: AB_94882
AQP4	Alomone Labs	Cat# AQP-004; RRID: AB_2039734
AT8	Thermo Fisher Scientific	Cat# MN1020; RRID: AB_223647
Cd68	BioLegend	Cat# 137001; RRID: AB_2044003
Dectin-1 (Clec7a)	InvivoGen	Cat# mabg-mdect-2; RRID: AB_2753143
FOXP2	Abcam	Cat# Ab16046; RRID: AB_2107107
GFAP	Synaptic System	Cat# 173 004; RRID: AB_10641162
GFAP	DAKO	Cat# Z033401-2; RRID: AB_10013382
HLA DR + DP + DQ	Abcam	Cat# Ab7856; RRID: AB_306142
Human APOE	Abcam	Cat# Ab52607; RRID: AB_867704
Human GFAP	BioLegend	Cat# 837202; RRID: AB_2565372
Human Nuclear Antigen	Millipore	Cat# MAB1281; RRID: AB_94090
IBA1	Synaptic Systems	Cat# 234 004; RRID: AB_2493179
IBA1	Synaptic Systems	Cat# 234 008; RRID: AB_2891288
Lycopersicon Esculentum Lectin	VectorLabs	Cat# DL-1178-1; RRID: AB_2336416
LTDA_Aβ capture antibody	Preman et al.^5^	Available from Drs. Bart de Strooper and Maarten Dewilde labs
Nestin	Abcam	Cat# Ab22035; RRID: AB_446723
NeuN	Synaptic Systems	Cat# 266-004; RRID: AB_2619988
P2Y12	Synaptic Systems	Cat# 476-011; RRID: AB_2924961
P2Y12	Sigma	Cat# HPA014518; RRID: AB_2669027
PAX6	Abcam	Cat# Ab5790; RRID: AB_305110
RFP	Rockland	Cat# 600-401-379; RRID: AB_2209751
RFP	Rockland	Cat# 200-301-379; RRID: AB_2611063
S100	Abcam	Cat# Ab868; RRID: AB_306716
S100b	Abcam	Cat# Ab52642; RRID: AB_882426
SOX2	Cell Signaling	Cat# 3579S; RRID: AB_2195767
Sulfo-TAG detection antibody	Preman et al.^5^	Availeble from Drs. Bart de Strooper and Maarten Dewilde labs
Vimentin	BD Biosciences	Cat# 550513; RRID: AB_393716
Anti-Mouse 488	Invitrogen	Cat# A21202; RRID: AB_141607
Anti-Mouse 594	Invitrogen	Cat# A21203; RRID: AB_2535789
Anti-Mouse 647	Invitrogen	Cat# A31571; RRID: AB_162542
Anti-Mouse biotinylated	Vector Laboratories	Cat# BA-9200; RRID: AB_2336171
Anti-Mouse HRP	DAKO	Cat# P0447; RRID: AB_2617137
Anti-Rabbit 488	Invitrogen	Cat# A21206; RRID: AB_2535792
Anti-Rabbit 594	Invitrogen	Cat# 21207; RRID: AB_141637
Anti-Rabbit biotinylated	Vector Laboratories	Cat# BA-1000; RRID: AB_2313606
Anti-Guinea Pig 647	Jackson ImmunoResearch	Cat# 706-175-148; RRID: AB_2340462

**Biological samples**		

Human brain samples	Fundacion CIEN	https://www.fundacioncien.es/

**Chemicals, peptides, and recombinant proteins**		

mTeSR1	StemcellTechnologies	Cat# 85850
Matrigel	Corning	Cat# 354230
Accutase	Stemcell Technologies	Cat# 07920
Thiazovivin (Tzv)	Millipore	Cat# 420220-M
LDN193189	Stemcell Technologies	Cat# 72147
SB431542	Stemcell Technologies	Cat# 72234
DMEM/F12	Invitrogen	Cat# 10565
N2	Invitrogen	Cat# 17502-048
B27-RA	Invitrogen	Cat# 12587-010
Rosette Selection Reagent	Stemcell Technologies	Cat# 05832
FGF2	Invitrogen	Cat# 100-18B-50UG
Tryple Express	Gibco	Cat# 12605028
Astrocyte medium	ScienCell	Cat# 1801
HB-EGF	Peprotech	Cat# 100-47
Rock inhibitor (Y-27632)	Cells guidance systems	Cat# Y-27632
Paraformaldehyde	Sigma	Cat# 441244
Phosphate-buffered saline (PBS) without calcium and magnesium *X*1 pH 7.4	VWR	Cat# 392-0442
Ethylene glycol	Fisher Scientific	Cat# 10532595
Glycerol	Sigma	Cat# G5516
RNasin (RNAse inhibitor)	Sigma	Cat# 3335402001
Tri-sodium citrate buffer	VWR	Cat# 27833.294
Donkey serum	Fisher Scientific	Cat# 11404228
Triton X-100	Fisher Scientific	Cat# 11471632
DAPI (4′,6-Diamidino-2-Phenylindole, Dilactate)	Fisher Scientific	Cat# 11530306
Thioflavin-S	Sigma	Cat# 1326-12-1
X-34	Merk	Cat# SML1954
Ethanol	VWR	Cat# 83813.41
NaOH (sodium hydroxide)	Sigma	Cat# S5881
Glycergel mounting media	DAKO	Cat# C0563
R-Universal Buffer	Aptum Biologics	Cat# AP0530
Tris-HCl UltraPure 1M pH 7.5 Buffer	Fisherbrand	Cat# 10123722
Autofluorescence Eliminator Reagent	Merck	Cat# 2160
Fluoromount-G mounting medium	ThermoFisher	Cat# 00-4958-02
Protease inhibitor cocktail	Roche	Cat# 5056489001
Tween 20	FisherBrand	Cat# 13464259

**Critical commercial assays**		

R-PLEX Human APOE Assay kit	Meso Scale Discovery	Cat# K1509MR-2
NYZ Total RNA isolation Kit	Nzytech	Cat# MB13402
Qubit RNA HS Assay Kit	Thermo Fisher Scientific	Cat# Q32855
SMARTer Stranded Total RNA-seq Kit v3 - Pico Input Mammalian	Takara Bio	Cat# 634489-634492
SMARTer RNA Unique Dual Index Kit 24U	Takara Bio	Cat# 634451
Agilent High Sensitivity DNA kit	Agilent Technologies	Cat# 5067-4626

**Deposited data**		

RNA-seq analyses of mouse transcripts in APOE4 AD vs. APOE3 AD chimeras	This paper	GSE335500

**Experimental models: Cell lines**		

Human: iPSC lines	TCW et al.^14^	Available from Drs. Alison Goate and Julia TCW labs

**Experimental models: Organisms/strains**		

Mouse: *APP PS1 Prkdc*^scid/scid^	Preman et al.^8^	N/A
Mouse: *App*^*NL-G-F*^*Rag2*^−/−^ mice	Balusu et al.^46^	Available from Dr. de Strooper lab

**Recombinant DNA**		

lentiGuide-Hygro-dTomato	Addgene	Cat# 99376

**Software and algorithms**		

LAS X	Leica Microsystems	Leica Application Suite X
Fiji/ImageJ	Fiji (ImageJ)	RRID: SCR_002285
Graphpad Prism 8	Graphpad Software	RRID:SCR_002798
R v4.3.3	R Project for Statistical Computing	RRID: SCR_001905
DESeq2 v1.44.0	Bioconductor	RRID: SCR_015687
FastQC v0.11.9	Babraham Bioinformatics	RRID: SCR_014583
Trimmomatic v0.39	Usadel Lab	RRID: SCR_011848
SortMeRNA v4.3.6	GitHub/Bioinformatics & Biostatistics Hub, Institut Pasteur	RRID: SCR_014402
HISAT2 v2.2.1	Center for Computational Biology, Johns Hopkins University	RRID: SCR_015530
featureCounts v2.0.6	Subread package	https://academic.oup.com/bioinformatics/article/30/7/923/232889?login=false
MGEnrichment	Jao & Ciernia et al.^35^	Available from Ciernia Lab/GitHub: ciernialab/MGEnrichmentApp

### Experimental model and study participant details

#### hiPSC lines

Eight hiPSC lines were generated from three *APOE E4/E4* carriers diagnosed with AD (Table S1) as described previously.^8^^,^^14^ hiPSCs were maintained in mTeSR1 plus medium (STEMCELL Technologies) on 6-well plates precoated with Matrigel (Corning). Medium was changed daily, and cells were routinely passaged when 80–90% confluent using Accutase (STEMCELL Technologies) supplemented with 10 μM Thiazovivin (Tzv, Millipore). All lines were characterized for pluripotency, capability to differentiate into different CNS cell types, routinely tested negative for Mycoplasma contamination, and G-banding karyotyping was confirmed normal.^8^^,^^14^ The identity of the hiPSC lines was confirmed by genetic identity-by-descent analysis (PI HAT) using Global Screening Array genotyping, demonstrating genetic concordance with the original donor fibroblasts, as previously described.^14^

#### Mice

We used two mouse models suitable for transplantations that were generated as described previously.^8^^,^^46^ Briefly, *APP PS1* tg/wt mice, which express the mutated *APP* (KM670/671NL) and *PS1* (L166P) under the control of the Thy1.2 promoter1.1,^47^ were crossed with immunodeficient NOD-SCID mice (NOD.CB17-Prkdc^scid^) that carry a single point mutation in the *Prkdc* gene.^48^ *APP PS1* tg/wt *Prkdc*^scid/+^ mice from the F1 generation were subsequently crossbred with NOD-SCID mice to generate *APP PS1* tg/wt *Prkdc*^scid/scid^ immunodeficient mice. Resulting *APP PS1* tg/wt *Prkdc*^scid/scid^ mice were then bred with NOD-SCID mice to generate either *APP PS1* tg/wt *Prkdc*^scid/scid^ (AD mice) or *APP PS1* wt/wt *Prkdc*^scid/scid^ (WT mice), which were used for transplantations and histopathological analyses. In addition, homozygous *App*^*NL-G-F*^ (*App*^*tm3.1Tcs*^) knock-in mice^49^ or homozygous *App*^*hu/hu*^ (*App*^*em1Bdes*^) with a humanized Aβ sequence (G676R, F681Y, R684H)^50^ were crossed with homozygous *Rag2*^−/−^ (*Rag2*^tm1.1Cgn^) knockout mice to obtain the *App*^*NL-G-F*^ *Rag2*^−/−^ genotype (AD mice) or *App*^*hu/hu*^ *Rag2*^−/−^ genotype (WT mice). Homozygous colonies were maintained, and crosses were conducted within the same genotype to obtain mice used for transplantations, transcriptomic analyses and ELISA analyses. Both female and male mice were used in experiments.

Animals were housed in groups of four per cage with *ad libitum* access to food and water in individually ventilated cages (IVC) within a specific pathogen-free (SPF) facility, where light/dark cycle and temperature were controlled. Experiments were performed on both male and female littermates. All rodent experiments were approved by the Ethics Committee for Animal Care and Use in Research (CEEA-UPV/EHU, reference number M20_2022_117) of the University of the Basque Country and were executed in compliance with the ethical regulation for animal research.

#### Human brain samples

Brain tissue samples from the lateral temporal cortex (Brodmann areas 21 and 22) of 6 patients with AD carrying the *APOE3/E3* alleles, 5 patients carrying the *APOE4/E4* alleles and 4 non-demented individuals were provided by the Biobank Banco de Tejidos CIEN (Table S4). They were processed following standard operating procedures with the appropriate approval of the Research Ethics Committee of the Instituto de Salud Carlos III (ISCIII, reference number CEI PI 48_2023) and with informed consent from all participants. Brain tissues were collected as described in previous studies,^51^ with an average postmortem interval (PMI) of 4.5 h. Briefly, after autopsy, the brains were fixed in 4% buffered formaldehyde for at least 4 weeks. Samples from the anterior entorhinal cortex and hippocampus were dissected coronally, dehydrated and embedded in paraffin. The paraffin blocks were microtomed at 5 μm, mounted on Flex IHC adhesive microscope slides (Dako), and dried at 55°C before storing. For neuropathological analysis, sections from all blocks were stained with anti-pTau (AT8), anti-Aβ (4G8) and with the Gallyas and the Campbell-Switzer silver techniques for detection of neurofibrillary changes and amyloid deposits.^52^ The postmortem diagnosis of AD neuropathological change was based upon the standardized consensus criteria, including staging of Aβ plaques based on Aβ immunohistochemistry,^52^ the Braak neurofibrillary tangle (NFT) stage based on pTau immunohistochemistry,^53^ and the frequency of neuritic plaques, which compose the ABC classification system.^54^ The study comprised 15 cases with an average age of 80 years.

### Method details

#### Generation of reporter hiPSC-derived astrocyte progenitors

hiPSCs plated in Matrigel coated plates and cultured in mTeSR1 medium were differentiated into neural progenitor cells (NPCs) by dual SMAD inhibition (0.1 μM LDN193189 and 10 μM SB431542) in embryoid bodies (EB) media (DMEM/F12 (Invitrogen, 10565) with 1*x* N2 (Invitrogen, 17502-048) and B27-RA (Invitrogen, 12587-010)). Rosettes were selected after 14 days *in vitro* (DIV) using Rosette Selection Reagent (StemCell Technologies) and additionally directed into forebrain NPCs using EB media supplemented with 20 ng/mL FGF2 (Invitrogen). Magnetic-activated cell sorting (Miltenyi Biotec) was used to enrich for the NPCs (CD271−/CD133+).^55^ NPCs were validated by immunocytochemistry using specific markers (SOX2, PAX6, FoxP2, and Nestin). Forebrain NPCs were dissociated using Tryple Express (Gibco, 12605028), seeded at a density of 1,000,000 cells per well in 12-well plates, transfected with the lentiGuide-tdTomato plasmid (Addgene #99376), and selected by hygromycin. Subsequently, purified fluorescent NPCs were seeded at a low density (15,000 cells/cm2) on Matrigel-coated plates and differentiated into astrocytes in astrocyte medium (ScienCell, 1801), as described previously.^26^ Astrocyte progenitors were cultured *in vitro* until DIV 40–44, their identity was confirmed by immunocytochemistry for astrocyte-specific markers, and they were used for transplantation experiments.

#### Transplantation of hiPSC-derived astrocyte progenitors

Transplantation experiments were performed on neonatal AD and WT mice at postnatal days P1-P4 following previously described protocols^8^ with minor modifications. In brief, hiPSC-derived astrocyte progenitor cells at DIV 44 were enzymatically dissociated using Accutase (STEMCELL Technologies, 7920), supplemented with HB-EGF (100−47, Peprotech) and Rock inhibitor (Y-27632, Cells guidance systems), and injected into the frontal cortex of AD or WT mice. Pups were cryoanesthetized, and approximately 200,000 cells were injected using Hamilton syringes into two locations within the forebrain: 1 mm posterior to bregma, 1.5 mm bilaterally from the midline, and 1.2 mm from the pial surface in *APP PS1 Prkdc* mice; and 1 mm and 2 mm posterior to bregma, 1.5 mm unilaterally from the midline, and 1.2 mm from the pial surface in *App Rag2*^−/−^ mice. After transplantation, the pups were allowed to recover under a heat lamp at 37°C and then returned to their home cages until reaching the weaning age. Grafted pups were monitored daily for a week.

#### Sample isolation from chimeric mice

For immunofluorescence (IF) analysis, mice at 5–6 months after transplantation were euthanized with CO_2_ and immediately perfused with phosphate-buffered saline followed by a 4% paraformaldehyde solution. Subsequently, the brain was extracted, postfixed in the same fixative solution overnight for up to 48 h and sectioned into 40 μm thick coronal sections using a Leica VT1000S vibratome. Brain sections were stored in cryoprotectant solution (30% ethylene glycol, 30% glycerol, 40% PBS) at −20°C until use.

For RNA and protein extraction, mice at 5–6 months after transplantation were anesthetized with 100 mg/kg ketamine (Fatro) and 10 mg/kg xylazine (Calier) and animals were euthanized using cervical dislocation. Brains were surgically removed and sliced into 1 mm thick coronal sections using a cold brain matrix. Sections were collected immediately into cold PBS (containing RNasin (0.2u/ul) in samples used for RNA extraction) and placed under a fluorescent dissection stereo microscope (Leica MZ10F). The fine dissection of the RFP-positive areas, the host tissue next to RFP-positive areas and the contralateral brain regions was performed. The collected samples were snap-frozen in liquid nitrogen and stored at −80 C until use.

#### Immunofluorescence in chimeric mice

IF staining on grafted brains was performed as previously^8^ using primary and secondary antibodies. Briefly, vibratome sections were washed three times with PBS to remove the residual storing solution. Antigen retrieval was performed by microwave boiling the sections in 10 mM tri-sodium citrate buffer (VWR) with Tween 20 (0.1%) at pH 6.0. After rinsing with PBS, the free-floating sections were placed in permeabilization/blocking buffer containing 5% serum, corresponding to the host species of the secondary antibody (Donkey) in PBST (PBS with 0.20% Triton X-100 in 1xPBS) for 1 h at room temperature. After the blocking step, primary antibodies diluted in the blocking solution were added to the sections and incubated overnight at 4°C. The next day, sections were washed three times for 5 min in PBST. Respective secondary antibodies were added, and sections were incubated for 2 h at room temperature. If appropriate, nuclei staining was performed using a specific anti-human Nuclear Antigen antibody (hNuclei) or DAPI (Sigma). Aβ plaques were visualized by staining with Thioflavin-S (1326-12-1, Sigma) or X-34 (SML1954, Merk); and the antibody 6C3 was also used to visualize Aβ plaques. For X-34 staining, brain sections were permeabilized with 0.25% Triton X-100 in PBS for half an hour, incubated in X-34 staining solution (10 μM X-34 diluted in 60% PBS (vol/vol), 40% ethanol (vol/vol), and 20 mM NaOH) for 20 min at room temperature, and rinsed in 40% ethanol/PBS solution and PBS containing 0.1% Triton X-100.^32^ For Thioflavin staining, brain sections were incubated with a filtered 0.05% aqueous Thioflavin solution in 50% ethanol for 5 min at RT, followed by gradual rinsing with 70%, 95% ethanol and water. Subsequently, the samples were washed three times for 5 min in PBST, allowed to dry at room temperature and mounted onto glass slides using the Glycergel mounting media (DAKO).

#### Immunofluorescence in human brain samples

Paraffin-embedded human sections (5 μm thick) were deparaffinized and rehydrated by immersion in xylene followed by incubations with alcohol solutions (100%, 96%, 75%, and 50% diluted in H_2_Odd) for 10 min each, and finally, three 5-min washes were performed with H_2_O. Samples were then boiled in R-Universal Buffer (Aptum Biologics) using Antigen retriever 2100 (Aptum Biologics) for 20 min and allowed to cool down for 20 min to promote epitope unmasking. After retrieval, samples were washed in TBST three times for 10 min and blocked in 4% BSA and Tween 0.1% solution in TBS for 1 h at RT. Following blocking, samples were incubated with primary antibodies diluted in the blocking solution overnight at RT. Then, samples were washed in TBST three times and incubated with fluorochrome-conjugated secondary antibodies diluted in the blocking solution for 1 h at RT. Samples were washed in TBST and incubated with a filtered 0.05% aqueous Thioflavin S solution in 50% ethanol for 5 min at RT, followed by gradual rinsing with 70% and 95% ethanol in TBS, and water. After that, samples were washed in TBST three times and immersed in 70% ethanol for 5 min before being treated with Autofluorescence Eliminator Reagent (Merck, 2160) according to the manufacturer’s instructions to reduce lipofuscin-like autofluorescence. Finally, sections were washed and mounted with Fluoromount-G mounting medium (Thermofisher).

#### Image acquisition and analyses

Confocal images were obtained using a Leica TCS STED CW SP8 confocal. For excitation, 405 nm, 488 nm, 561 nm, 638 nm laser lines were used. All the images were obtained using a 20*x* (0.75 NA) or 40*x* oil (1.4 NA) objective lens, and z stack series of images of the area of interest were acquired using the LAS X software. 9–10 Z-stacks with a spacing of 1 μm in chimeric brain sections and 6 Z-stacks with a spacing of 1 μm in human brain sections were obtained. All images were acquired using identical acquisition parameters such as 16-bit, 1024 × 1024 quality, and images were processed in the FIJI/ImageJ software. Images of z series stacks were converted to maximum intensity projections for quantifications, unless otherwise specified. Except in apoE analysis, all comparisons are between ipsilateral regions hosting hAstrocytes and their corresponding contralateral areas in the same cortical section.

##### Analyses of cell integration

For analyses of cell integration, brain sections were stained with antibodies against RFP and human nuclear antigen (hNuclei). The number of hNuclei+ and RFP+ cells was manually quantified from IF images of astrocytes derived from the eight hiPSC lines used in this study (Table S1) in both WT and AD chimeric mice. Cell numbers were corrected for the sectioning series (1:6) to generate estimates of the total number of integrated hNuclei^+^ and RFP^+^ cells per animal (Table S2). Integration values represent estimates rather than exact counts.

##### Quantification of human APOE (hAPOE) intensity

To study hAPOE within hAstrocytes, sum projections of 40*x* images were used to measure mean pixel intensity per area in *APOE3* and *APOE4* hAstrocytes in WT and AD mice. To quantify extracellular hAPOE, the mean intensity of extracellular hAPOE was measured along 100 μm from the soma of hAstrocytes, thus obtaining the intensity of extracellular hAPOE relative to the area occupied by hAstrocytes. For the analysis of hAPOE in Aβ plaques, mean pixel intensity was measured in plaques within 100 μm of hAstrocytes. The antibody used for these experiments is specific for human APOE and does not recognize mouse APOE. The contralateral hemisphere served as an internal negative control for human APOE staining. Therefore, in the contralateral hemisphere, no signal above background was detected when using identical staining conditions and acquisition settings.

##### Quantification of Aβ burden

Images were obtained with the 3DHistech Pannoramic MIDI II Slide Scanner. For quantification of the Aβ burden in regions with hAstrocytes and contralateral regions, the region with hAstrocytes and a mirror region in the contralateral hemisphere were manually selected. The % area occupied by Aβ plaques, as well as the number and size of Aβ plaques were quantified using the RenyiEntropy (for Thioflavin) or the Otus (for 6C3) algorithm in Fiji/ImageJ.

##### Analysis of Aβ plaque morphology

For the analysis of Aβ plaque morphology, the circularity and the Angular Second Moment (ASM) were calculated in maximum intensity projections of the z stack images (40*x*). Plaques of the same size were quantified in ipsilateral and contralateral regions and a fixed threshold of 40 was used. ASM of the gray-scale co-occurrence matrix was calculated as previously^5^ using the plugin GLCM texture of Fiji/ImageJ to evaluate amyloid plaque compactness. ASM serves as an indicator of local image homogeneity, with a higher value indicative of a more homogenous plaque structure with fewer transitions between light and dark areas.

##### Quantification of the plaque-associated microglia, Tau and dystrophic neurites

For quantification of the plaque-associated microglia (Iba-1, Clec7a and P2y12 signals), Tau (AT8) and dystrophic neurites (APP), images (40*x*) of z series stacks were converted to Fiji/ImageJ maximum intensity projections, and plaques with hAPOE within a maximum distance of 100 μm from the hAstrocytes were selected on each image. A 30 μm ring from the center of mass of the plaques was drawn, except in the case of P2y12 signals in which a 40 μm ring was drawn. The area occupied by Iba-1 was quantified using the Li algorithm in Fiji/ImageJ. A uniform threshold, with fixed values within each animal, was then applied to measure the areas occupied by P2Y12 (20 ± 5), Clec7a (20 ± 5), Tau (40 ± 5), and dystrophic neurites (40 ± 5) within the 30 or 40 μm ring. In brief, plaques were identified with fixed threshold, and a circular area with a radius of 30 or 40 μm was generated from the center of mass of the plaque in the z stack images. Iba-1, Clec7a, P2y12, AT8, or APP-positive area within the 30 or 40 μm ring was measured, normalized against the total expanded area around the plaque, and represented as percentage positive area within the 30 or 40 μm ring. The number of AT8-positive puncta within the 30 μm ring was quantified using Analize Particles in Fiji/ImageJ. Plaques of the same size were quantified in ipsilateral and contralateral regions.

##### Quantification of microglia coverage of the plaques

For the analysis of microglia coverage, the same images and plaques used to quantify plaque-associated microglia were analyzed. A maximum intensity projection of the Iba1 and plaque channels was generated. The percentage of plaque area occupied by microglia was quantified by performing a colocalization analysis in Fiji/ImageJ, using the Li algorithm to identify the Iba1-positive area, while the plaque ROI was defined by applying a fixed threshold of 40. Once the percentage of plaque area occupied by microglia was calculated, the ipsilateral values were normalized to their corresponding contralateral results.

##### Quantification of Cd68 in microglia

For quantification of microglial Cd68 signals, images (63*x*) of z series stacks were converted to Fiji/ImageJ maximum intensity projections, and plaques with hAPOE within a maximum distance of 100 μm from the hAstrocytes were selected on each image. Plaques were identified with the MaxEntropy algorithm threshold and a 30 μm ring from the center of mass of the plaques was drawn. A threshold of 20 ± 5 was used to select Iba-1 signals within the 30 μm ring, and the Li algorithm was then applied to measure the percentage Cd68 area within the Iba-1 area. Plaques of the same size were quantified in ipsilateral and contralateral regions.

##### Quantification of total microglia and Aβ burden close to hAstrocytes

For the analysis of total microglia and Aβ plaque burden close to hAstrocytes, images (20*x*) of z series stacks were acquired at the periphery of the regions with hAstrocytes and converted to Fiji/ImageJ maximum intensity projections. The area occupied by microglia or Aβ plaques was quantified using a uniform threshold (35 ± 5) with fixed values within each animal. Briefly, both plaques and microglia were identified with fixed threshold, and the positive area was measured, normalized against the total area, and represented as percent positive area.

##### Quantification of Aβ burden and APOE intensity in human cases

Individual plaques larger than 350 μm were segmented using an interactive machine learning approach, implemented through ImageJ and the Labkit plugin.^56^^,^^57^ Through this segmentation of the plaque channel, the individual plaque ROIs, the amount of Aβ, the number and size of plaques were obtained. These ROIs were also used to analyze the intensity of APOE within and around each Aβ plaque using the sum projection of the APOE channel. To measure APOE surrounding the plaques, the plaque ROI was enlarged by 10 μm, and the original plaque ROI was subtracted, leaving only the area within a 10 μm distance around the plaque.

#### RNA extraction and quality control

Total RNA was extracted from 5 mg of brain tissue using the NYZ Total RNA isolation KIT (MB13402, nzytech). RNA quantity was assessed using the Qubit RNA HS Assay Kit (Thermo Fisher Scientific, Cat.#Q32855). RNA integrity was confirmed using the Agilent 2100 Bioanalyzer with Agilent RNA 6000 Pico Chips. All samples exhibited high RNA Integrity Number (RIN) values, generally ranging from 7.8 to 9.5.

#### Library preparation and sequencing

Libraries were prepared using 10ng of total RNA input with the SMARTer Stranded Total RNA-seq Kit v3 - Pico Input Mammalian (Takara Bio, Cat. Nos. 634489–634492) and the SMARTer RNA Unique Dual Index Kit 24U (Takara Bio, Cat. No. 634451), following the manufacturer’s user manual. Their size distribution was verified on an Agilent 2100 Bioanalyzer using Agilent High Sensitivity DNA kit (Agilent Technologies, Cat. # 5067-4626). Libraries were sequenced on an Illumina NovaSeq 6000 platform to generate 150 bp paired-end reads.

#### RNA-seq preprocessing, alignment and quantification

Raw FASTQ files underwent initial quality assessment with FastQC v0.11.9 and reads were trimmed and filtered using Trimmomatic v0.39. Ribosomal RNA (rRNA) was depleted using SortMeRNA v4.3.6 and non-rRNA reads were then aligned with HISAT2 v2.2.1 to a mouse (GENCODE M. musculus GRCm39 vM36) reference genome. Finally, gene counts were quantified using Subread’s featureCounts v2.0.6. For downstream analysis mouse read count matrices were analyzed, achieving means of 25,973,659 mouse reads per sample.

#### Differential expression analysis

Differential expression (DE) analysis was carried out using the DESeq2 package v1.44.0 in R v4.3.3 to identify genes significantly altered between AD mice with *APOE4* astrocytes and AD mice with *APOE3* astrocytes. To account for technical variation, the statistical model included both RNA Integrity Number (RIN) and Total RNA input as covariates. DE results are available in Table S5.

#### Microglia-specific enrichment analysis

To identify cellular processes and signaling pathways specifically associated with microglial response, we performed enrichment analysis on the resulting differentially expressed genes using the *MGEnrichment* online tool,^35^ an Enrichment analysis tool for bulk RNA-seq data to identify microglial-associated changes. This tool compares the observed expression profile of a gene set (e.g., *APOE4* AD vs. *APOE3* AD DEGs) against predefined microglial gene signatures derived from a curated database of studies with microglia-relevant gene list. Specifically, we focused on gene signatures associated with microglia development, general microglial function, and inflammation. This allows for quantitative assessment of the activation state and specific pathway changes within the microglia population, even in bulk tissue RNA-seq data. Enrichment results are available in Table S5.

#### Protein extraction and MSD-ELISA

Protein extraction was carried out following a protocol previously described.^5^ Briefly, frozen brain tissue was homogenized in PBS with a protease inhibitor cocktail (Roche #5056489001), using FastPrep (MP Biomedicals). The homogenate was then centrifuged at 5,000 rcf for 5 min at 4°C and the supernatant was ultra-centrifuged at 55,000 rpm (Rotor TLA-110, Beckman Optima TLX) for 60 min at 4°C. The supernatant from the ultracentrifugation was used as the PBS-soluble fraction. To obtain guanidinium chloride (GuHCl) based extraction, the pellet was resuspended in 6 M GuHCl, 50 mM Tris-HCl (pH 7.6) and supplemented with cOmplete protease inhibitor cocktail (Roche) and sonicated using a micro-tip for 30 s at 10% amplitude (Branson) and then incubated for 1 h at 25°C. Finally, the sample was ultracentrifuged at 70,000 rpm (Rotor TLA-110, Beckman Optima TLX) and the supernatant was diluted to 0.1 M GuHCl and used as guanidinium-soluble fraction.

Aβ42 and hAPOE levels were quantified on MSD 96-well plates using MSD-ELISA (Meso Scale Discovery) as previously described.^5^ For Aβ42, standard 96-well SECTOR plates (MSD #L15XA-3) were coated with LTDA_Aβ42 capture antibody (mouse monoclonal antibody against Aβ_42_ generated in collaboration with Bart De Strooper and Maarten Dewilde labs) overnight at 4°C. The next day, the plates were washed with PBS 0.05% Tween 20 and blocked using PBS with 1% casein for 4 h at RT. Synthetic dilutions of human Aβ_42_ were used as standards which, along with the samples, were preincubated with LTDA_hAβN labeled with a sulfo-TAG detection antibody (mouse monoclonal antibody against the N-terminal sequence of human Aβ, generated in collaboration with Bart de Strooper and Maarten Dewilde labs), in casein buffer for 5 min at RT. The blocked assay plate was washed five times with PBS 0.05% Tween 20, and the sample and detection antibody mix were added and incubated at 4°C. After overnight incubation the measurements were taken. The plate was then washed with PBS, and 2*x* Read T buffer (MSD) was added before reading the plate on an MSD Sector Imager 2400A. For hAPOE, the R-PLEX Human APOE Assay kit (MSD #K1509MR-2) was used. Briefly, GOLD 96-well Small Spot Streptavidin SECTOR plates (MSD) were coated with biotinylated capture antibody for 1 h at RT. After washing the plates with PBS 0.05% Tween 20, the standards and samples diluted in assay buffer were added and incubated for 1 h at RT. Finally, the detection antibody was added and incubated for 1 h at RT. After the incubation, the plate was rinsed and GOLD Read Buffer B (MSD) was added, and plates were read on an MSD Sector Imager 2400 A as before.

### Quantification and statistical analysis

Statistical analyses were carried out using GraphPad Prism 8 software. All data are shown as mean ± S.E.M (Standard error of the mean) with sample size and the statistical test used indicated in the figure captions. When comparing the means of two groups for a single variable, paired or unpaired two-tailed t-tests were conducted. For comparisons involving more than two groups for a single variable, a two-way ANOVA followed by Holm-Sidak’s multiple comparison test was employed. Adjusted *p* < 0.05 (^∗^), *p* < 0.01 (^∗∗^), *p* < 0.001 (^∗∗∗^) and *p* < 0.0001 (^∗∗∗∗^) were considered significant, and all significant *p*-values were included in Figures or noted in Figure legends.

## Supplemental information

Document S1. Figures S1–S8 and Tables S1–S4 and S6

Table S5. Summary of differentially expressed genes (DEGs) and MG enrichment in the mouse tissue from *APOE4* vs. *APOE3* chimeric mice

Document S2. Article plus supplemental information
