# Supplementary material for: APOE3 and APOE4 human astrocytes differentially modulate Alzheimer’s disease pathology and microglial responses in chimeric mice
Source: Cell Rep. 2026 Aug 8;45(8):117803. doi: 10.1016/j.celrep.2026.117803 (PMC13506673; doi:10.1016/j.celrep.2026.117803)
Supplement: Supplementary file 1 [file mmc1.pdf]

Cell Reports, Volume *45*

Supplemental information

APOE3 and *APOE4* human astrocytes differentially modulate Alzheimer's disease pathology and microglial responses in chimeric mice

Joan Cruz-Sese, Marta Mirón-Alcala, María Alfonso-Triguero, Jon Olalde, Leire Ruiz, Nuria Galbis-Gramage, Lorea Cortes, Laura Escobar, Pranav Preman, An Snellinx, Takashi Saito, Takaomi C. Saido, Laura Saiz-Aúz, Alberto Rábano-Gutiérrez, Ana M. Aransay, Ibai Diez, Julia Tcw, Alison Goate, Bart De Strooper, Elena Alberdi, and Amaia M. Arranz

Figure S1

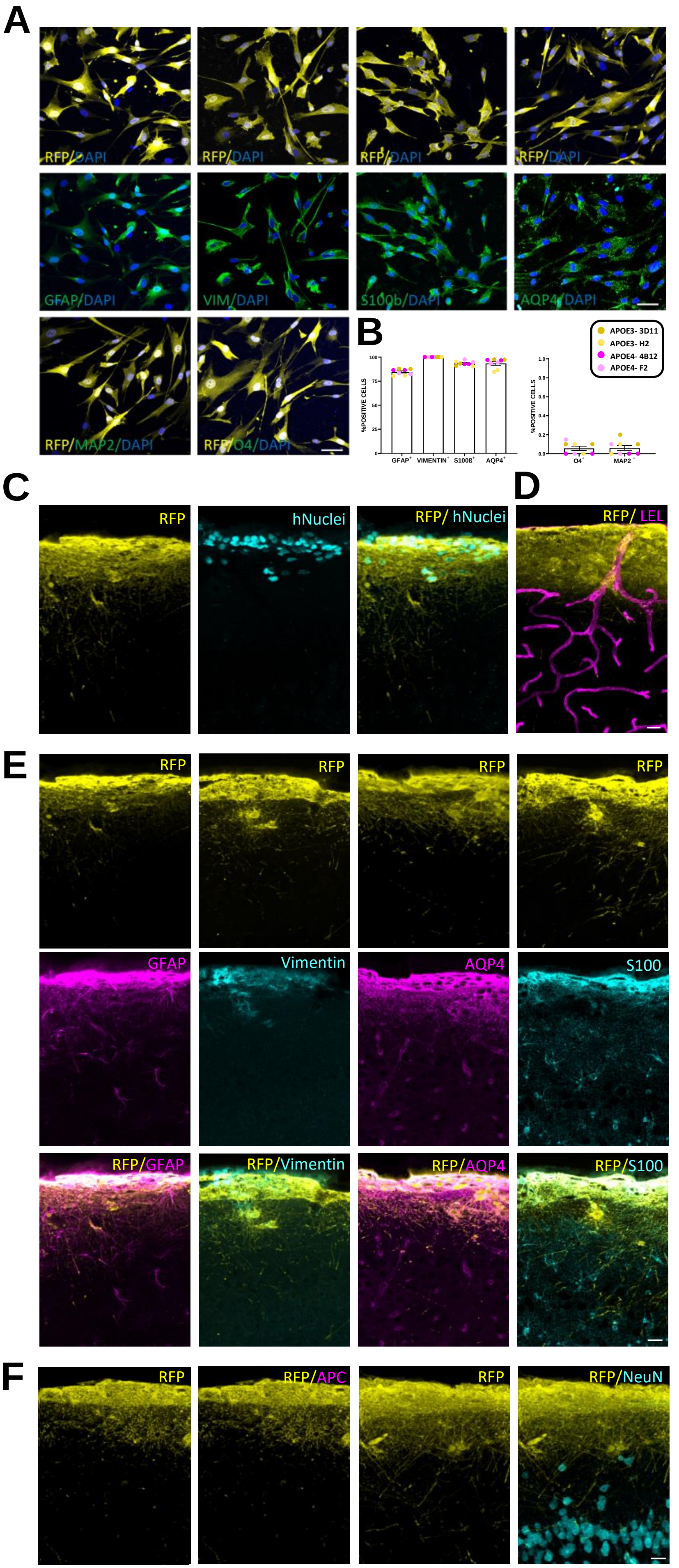
A
RFP/DAPI
RFP/DAPI
RFP/DAPI
RFP/DAPI
GFAP/DAPI
VIM/DAPI
S100b/DAPI
AQP4/DAPI
RFP/MAP2/DAPI
RFP/O4/DAPI
B
APOE3- 3D11
APOE3- H2
APOE4- 4B12
APOE4- F2
%POSITIVE CELLS
GFAP+
VIMENTIN+
S100B+
AQP4+
O4+
MAP2+
C
RFP
hNuclei
RFP/ hNuclei
D
RFP/ LEL
E
RFP
RFP
RFP
RFP
GFAP
Vimentin
AQP4
S100
RFP/GFAP
RFP/Vimentin
RFP/AQP4
RFP/S100
F
RFP
RFP/APC
RFP
RFP/NeuN

Figure S1. Characterization of hiPSC-derived astrocytes in culture and in vivo, related to Figure 1.
(A) Representative immunofluorescence images of human astrocytes (RFP, red) expressing the astrocyte-specific markers GFAP, Vimentin, AQP4 and S100β (green), and showing no expression of markers of oligodendrocytes (O4, green) or neurons (MAP2, green). Nuclei are counterstained with DAPI (blue). Astrocytes from the H-2 line are shown. Scale bars: 50 µm. (B) Percentage cells positive for astrocyte, neuron, and oligodendrocyte markers at DIV44. Data are represented as mean ± SEM (n=4 hPSC lines, 2 differentiations per line).
(C) Representative image of hAstrocytes (RFP, yellow) expressing the human nuclei marker (hNuclei, cyan) in Layer I of the cortex of a coronal brain section of a chimeric mouse 5 months after transplantation.
(D) Engrafted hAstrocytes (RFP, yellow) interact with blood vessels (LEL, magenta).
(E) Engrafted hAstrocytes (RFP, yellow) express main markers of astrocytes such as GFAP (magenta), Vimentin (cyan), AQP4 (magenta), and S100β (cyan).
(F) Engrafted hAstrocytes (RFP, yellow) show no expression of markers of oligodendrocytes (APC, magenta) or neurons (NeuN, cyan) 5 months after transplantation. Scale bars: 20 µm.

Figure S2

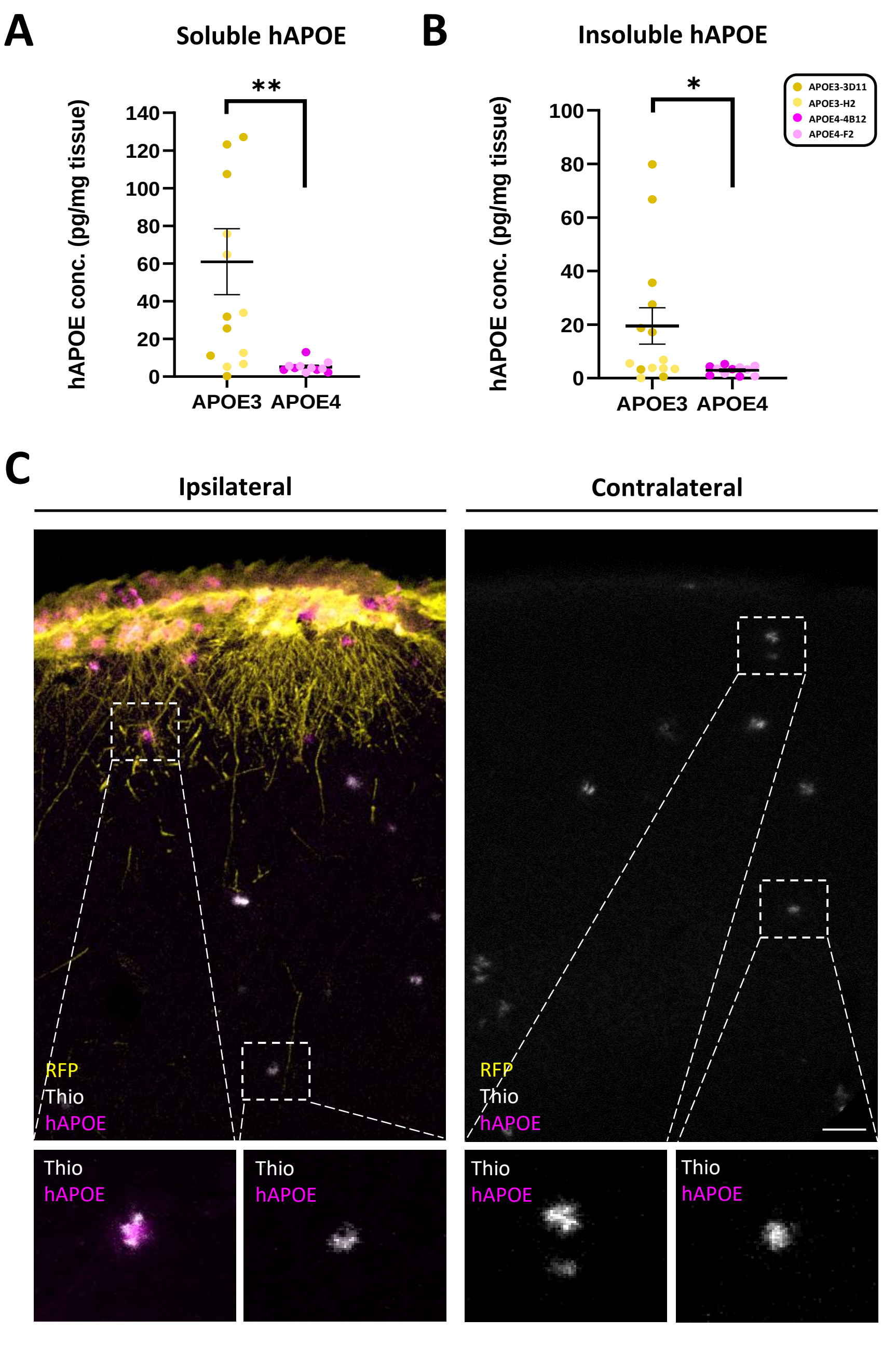

Figure S2. hAstrocytes express hAPOE that binds to Aβ plaques, related to Figure 2 and Figure 3.
(A) MSD-ELISA quantification of hAPOE protein levels in soluble fractions from cortical regions with *APOE3* and *APOE4* hAstrocytes in AD mice (*App*$^{NL\text{-}G\text{-}F}$ *Rag2*$^{-/-}$; n=14 mice with *APOE3* and n=11 mice with *APOE4* hAstrocytes).
(B) MSD-ELISA quantification of hAPOE protein levels in insoluble fractions from cortical regions with *APOE3* and *APOE4* astrocytes in AD mice (*App*$^{NL\text{-}G\text{-}F}$ *Rag2*$^{-/-}$; n=14 mice with *APOE3* and n=11 mice with *APOE4* hAstrocytes). Data are represented as mean ± SEM, unpaired t-test; ns = not significant, * = p < 0.05, ** = p < 0.01, *** = p < 0.001.
(C) General overviews of the ipsilateral and contralateral cortical hemispheres of chimeric mice showing the presence of hAPOE (magenta) within the plaques (Thio, white) close to hAstrocytes (RFP, yellow), while no hAPOE is detected within the plaques far from hAstrocytes or in the contralateral hemisphere. Enlarged images of the inserts are shown in the bottom panel. Scale bar: 50 μm.

Figure S3

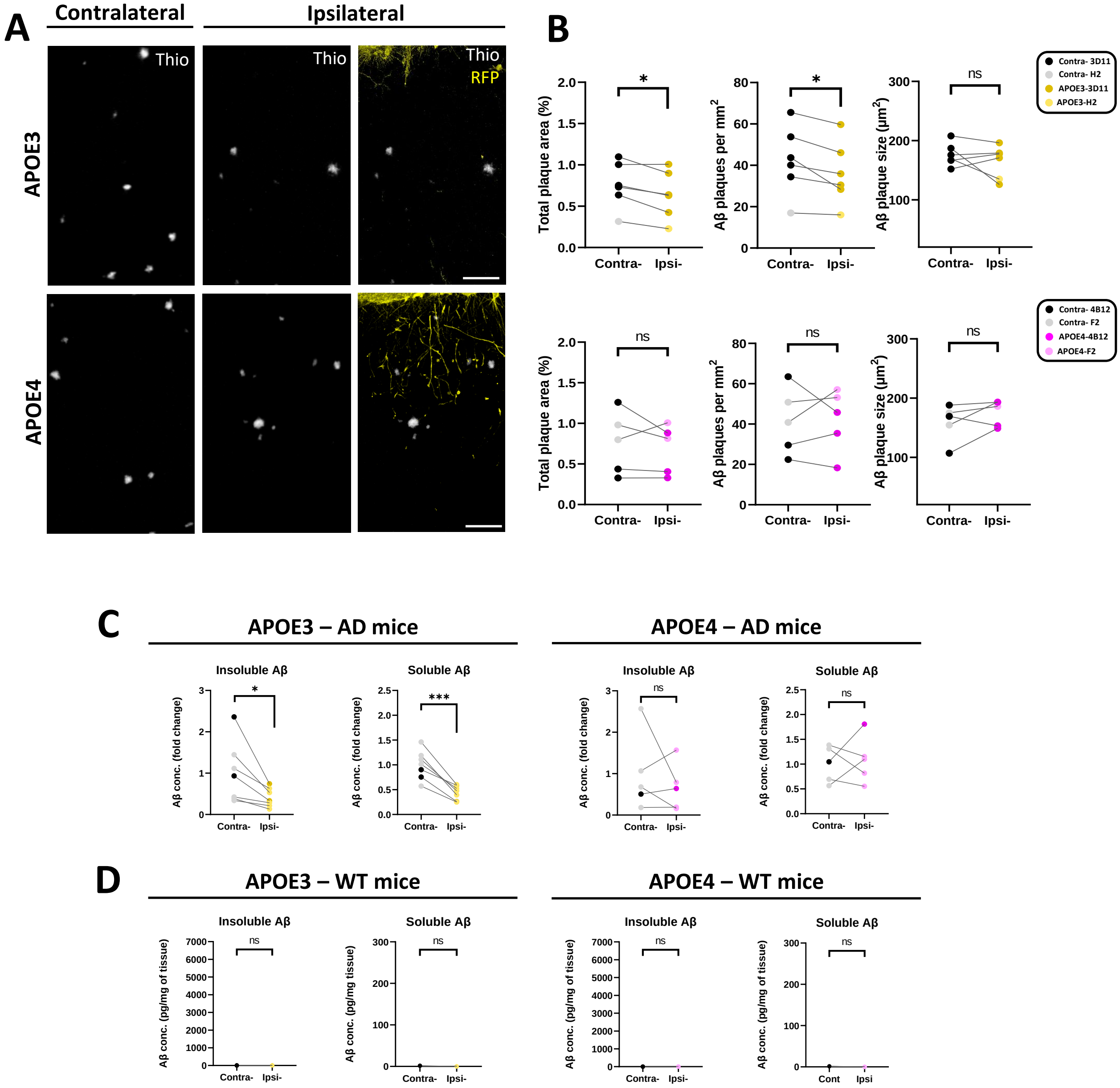

Figure S3. *APOE3* and *APOE4* hAstrocytes and hAPOE differentially affect overall Aβ burden, related to Figure 4.

(A) Representative images of the cortex of 5-6 month old AD chimeric mice stained for hAstrocytes (RFP, yellow) and fibrillar Aβ (Thio, white). Both contralateral and ipsilateral cortices hosting *APOE3* and *APOE4* hAstrocytes are shown. Scale bars: 100 μm.

(B) Quantification of Aβ burden, number of plaques per area and plaque size in contra- and ipsilateral cortices (*APP PS1 Prkdc*$^{scid/scid}$; n=6 mice with *APOE3* and n=5 mice with *APOE4* hAstrocytes). Data are represented as mean ± SEM, Paired t-test; ns = not significant, * = $p < 0.05$, ** = $p < 0.01$, *** = $p < 0.001$.

(C, D) MSD-ELISA quantification of Aβ levels in insoluble and soluble fractions from cortical regions with *APOE3* and *APOE4* hAstrocytes and contralateral areas in AD mice (C) and WT mice (D) (*App*$^{NL-G-F}$ *Rag2*$^{-/-}$; n=7 AD mice with *APOE3* and n=5 AD mice with *APOE4* hAstrocytes; n=10 WT mice with *APOE3* and n=8 WT mice with *APOE4* hAstrocytes). Data are represented as mean ± SEM, unpaired t-test; ns = not significant, * = $p < 0.05$, ** = $p < 0.01$, *** = $p < 0.001$.

Figure S4

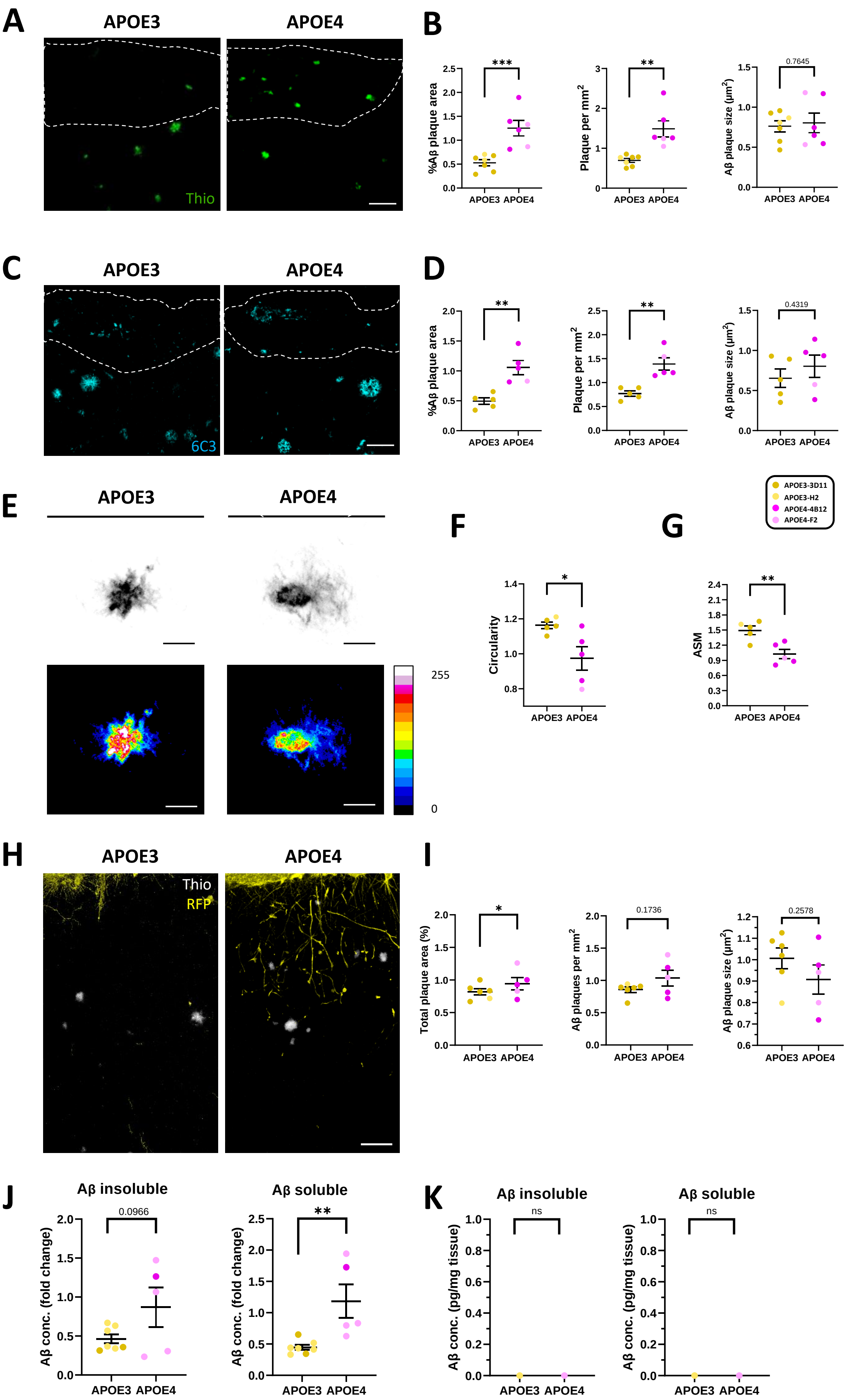
A
APOE3
APOE4
Thio
B
%Aβ plaque area
***
Plaque per mm²
**
Aβ plaque size (μm²)
0.7645
APOE3 APOE4
C
APOE3
APOE4
6C3
D
%Aβ plaque area
**
Plaque per mm²
**
Aβ plaque size (μm²)
0.4319
APOE3 APOE4
APOE3-3D11
APOE3-H2
APOE4-4B12
APOE4-F2
E
APOE3
APOE4
255
0
F
Circularity
*
G
ASM
**
APOE3 APOE4
H
APOE3
APOE4
Thio
RFP
I
Total plaque area (%)
*
Aβ plaques per mm²
0.1736
Aβ plaque size (μm²)
0.2578
APOE3 APOE4
J
Aβ insoluble
0.0966
Aβ soluble
**
Aβ conc. (fold change)
APOE3 APOE4
K
Aβ insoluble
ns
Aβ soluble
ns
Aβ conc. (pg/mg tissue)
APOE3 APOE4

Figure S4. *APOE3* and *APOE4* hAstrocytes differentially affect Aβ pathology, related to Figure 4.
(A) Representative images of the ipsilateral cortex of AD chimeric mice with *APOE3* or *APOE4* hAstrocytes 5-6 months after transplantation stained for fibrillar Aβ (Thio, green). Images are taken from Figure 4. Scale bars: 50 μm.
(B) Quantification of Aβ burden, number of plaques per area and plaque size in cortex regions hosting *APOE3* vs *APOE4* hAstrocytes (*APP PS1 Prkdc*$^{scid/scid}$; n=7 mice with *APOE3* and n=6 mice with *APOE4* hAstrocytes).
(C) Representative images of the ipsilateral cortex of AD chimeric mice with *APOE3* or *APOE4* hAstrocytes 5-6 months after transplantation stained for total Aβ (6C3, magenta). Images are taken from Figure 4. Scale bars: 50 μm.
(D) Quantification of Aβ burden, number of plaques per area and plaque size in cortex regions with *APOE3* vs *APOE4* hAstrocytes (*APP PS1 Prkdc*$^{scid/scid}$; n=5 mice with *APOE3* and n=5 mice with *APOE4* astrocytes).
(E) Representative images of X34-positive Aβ plaque conformation in grayscale and heat-density map based on intensity (red represents the highest intensity and blue is the lowest intensity) in cortices with *APOE3* and *APOE4* hAstrocytes. Images are taken from Figure 4. Scale bars: 10 μm.
(F, G) Quantification of circularity (F) and ASM degree of compactness (G) of X34-positive plaques in cortices with *APOE3* vs *APOE4* hAstrocytes (*APP PS1 Prkdc*$^{scid/scid}$; n=5 mice with *APOE3* and n=5 mice with *APOE4* astrocytes).
(H) Representative images of the cortex of 5-6 month old AD chimeric mice stained for hAstrocytes (RFP, yellow) and fibrillar Aβ (Thio, white). Cortices hosting *APOE3* and *APOE4* hAstrocytes are shown. Images are taken from Figure S3. Scale bars: 100 μm.
(I) Aβ burden, number of plaques per area and plaque size in cortices with *APOE3* vs *APOE4* hAstrocytes (*APP PS1 Prkdc*$^{scid/scid}$; n=6 mice with *APOE3* and n=5 mice with *APOE4* astrocytes).
(J, K) MSD-ELISA quantification of Aβ levels in insoluble and soluble fractions from cortical regions with *APOE3* vs *APOE4* hAstrocytes in AD mice (J) and WT mice (K) (*App*$^{NL\text{-}G\text{-}F}$ *Rag2*$^{-/-}$; n=7 AD mice with *APOE3* and n=5 AD mice with *APOE4* astrocytes; n=10 WT mice with *APOE3* and n=8 WT mice with *APOE4* astrocytes). Data are represented as mean ± SEM, unpaired t-test; ns = not significant, * = p < 0.05, ** = p < 0.01, *** = p < 0.001.

Figure S5

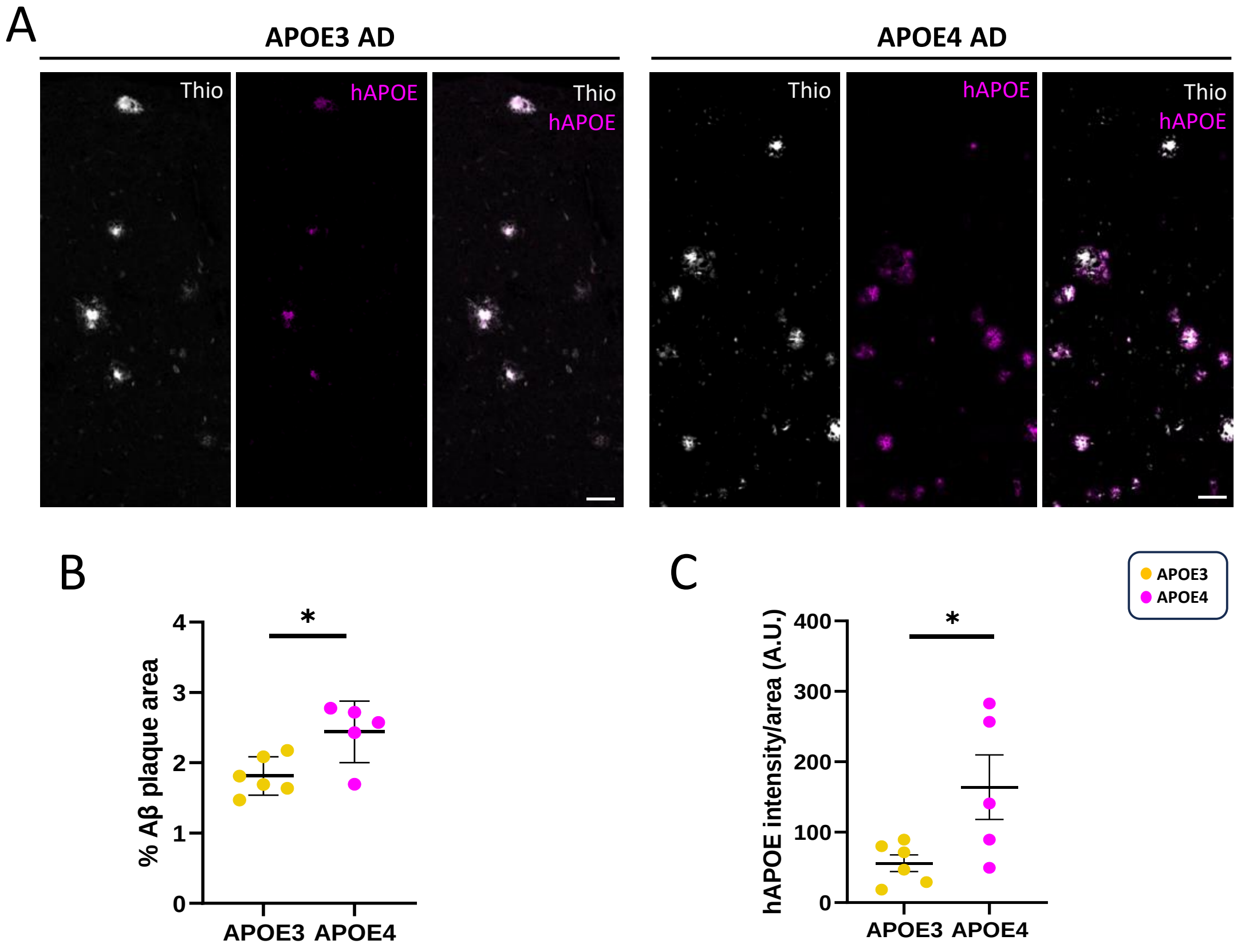

Figure S5. Human brains from *APOE4* patients with AD exhibit increased Aβ load, related to Figure 3 and Figure 4.

(A) Representative images of the lateral temporal cortex of patients with AD carrying *APOE3* or *APOE4* alleles stained for hAPOE (magenta) and fibrillar Aβ (Thio, white). Scale bars: 50 µm.

(B) Quantification of the Aβ plaque burden, and

(C) the mean intensity per area of plaque-associated hAPOE (n=6 patients carrying *APOE3/E3* alleles and n=5 patients with *APOE4/E4* alleles). Data are represented as mean ± SEM, unpaired t-test; ns = not significant, * = $p < 0.05$, ** = $p < 0.01$, *** = $p < 0.001$.

Figure S6

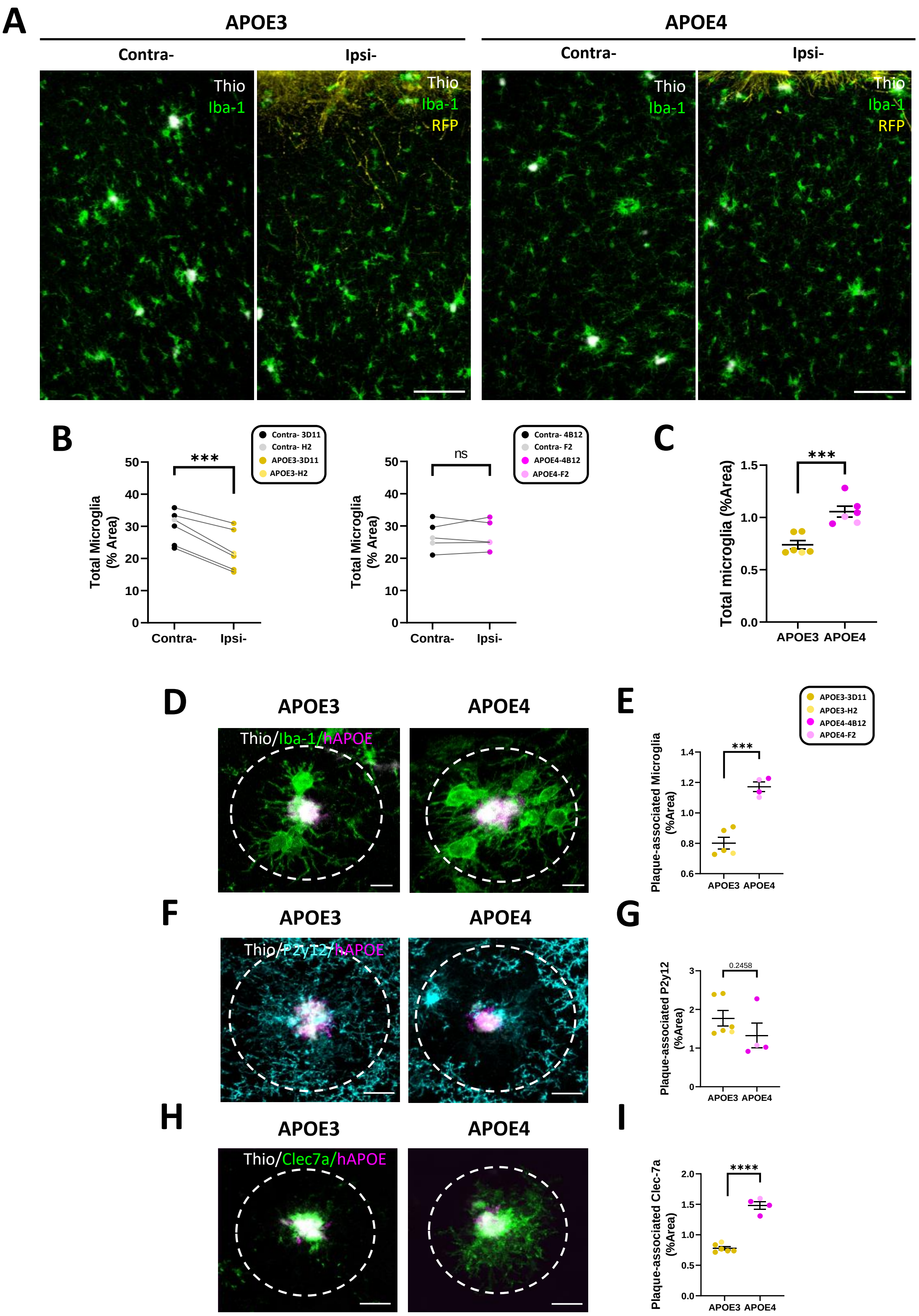
A
APOE3
APOE4
Contra-
Ipsi-
Thio
Iba-1
RFP
B
Total Microglia (% Area)
***
ns
Contra- 3D11
Contra- H2
APOE3-3D11
APOE3-H2
Contra- 4B12
Contra- F2
APOE4-4B12
APOE4-F2
C
Total microglia (%Area)
D
Thio/Iba-1/hAPOE
E
Plaque-associated Microglia (%Area)
F
Thio/P2y12/hAPOE
G
Plaque-associated P2y12 (%Area)
0.2458
H
Thio/Clec7a/hAPOE
I
Plaque-associated Clec-7a (%Area)
****

Figure S6. hAstrocytes and hAPOE modulate microglial responses in an isoform-dependent manner, related to Figure 5.

(A) Representative images of total microglia (Iba-1, green) in contralateral and ipsilateral cortices of AD chimeras hosting *APOE3* and *APOE4* hAstrocytes (RFP, yellow) 5-6 months after transplantation. Aβ plaques are stained with Thioflavin S (Thio, white). Scale bars: 100 μm.

(B) Percentage area covered by total microglia close to hAstrocytes in AD chimeras (*APP PS1 Prkdc*$^{scid/scid}$; n=6 mice with *APOE3* and n=5 mice with *APOE4* astrocytes). Data are represented as mean ± SEM, paired t-test.

(C) Percentage area covered by total microglia close to hAstrocytes in AD chimeras with *APOE3* vs *APOE4* hAstrocytes (*APP PS1 Prkdc*$^{scid/scid}$; n=6 mice with *APOE3* and n=5 mice with *APOE4* astrocytes).

(D) Representative images showing Iba-1 (green) positive microglia around Aβ plaques (Thio, white) with or without human APOE (hAPOE, magenta) in cortices of AD chimeras hosting *APOE3* and *APOE4* hAstrocytes 5-6 months after transplantation. Images are taken from Figure 5. Scale bars: 10 μm.

(E) Percentage area covered by Iba-1 positive microglia around Aβ plaques in *APOE3* vs *APOE4* AD chimeras (*APP PS1 Prkdc*$^{scid/scid}$; n=5 mice with *APOE3* and n=4 mice with *APOE4* astrocytes).

(F) Representative images of P2y12 (cyan) positive microglia cells surrounding Aβ plaques (Thio, white) with and without human APOE (hAPOE, magenta) in cortices of AD chimeras with *APOE3* and *APOE4* hAstrocytes 5-6 months after transplantation. Images are taken from Figure 5. Scale bars: 10 μm.

(G) Percentage area covered by P2y12 positive microglia around plaques (*APP PS1 Prkdc*$^{scid/scid}$; n=6 mice with *APOE3* and n=4 mice with *APOE4* astrocytes).

(H) Representative images of Clec7a (green) positive microglia cells surrounding Aβ plaques (Thio, white) with and without human APOE (hAPOE, magenta) in cortices of AD chimeras hosting *APOE3* and *APOE4* hAstrocytes 5-6 months after transplantation. Images are taken from Figure 5. Scale bars: 10 μm.

(I) Percentage area covered by Clec7a positive microglia around plaques (*APP PS1 Prkdc*$^{scid/scid}$; n=6 mice with *APOE3* and n=4 mice with *APOE4* astrocytes). Data are represented as mean ± SEM, unpaired t-test; ns = not significant, * = $p < 0.05$, ** = $p < 0.001$, *** = $p < 0.0001$.

Figure S7

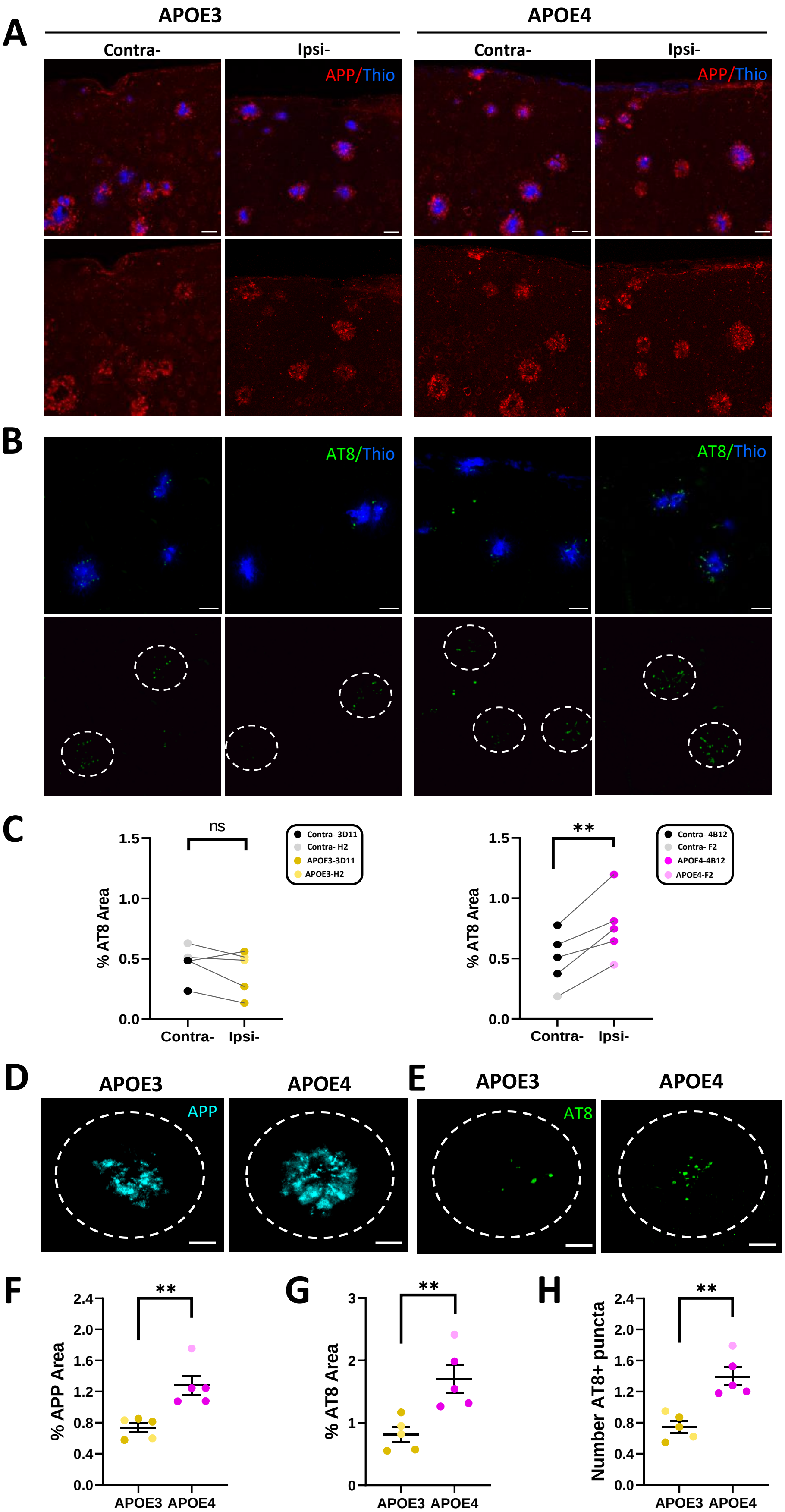
A
APOE3
APOE4
Contra-
Ipsi-
APP/Thio
B
AT8/Thio
C
ns
**
% AT8 Area
1.5
1.0
0.5
0.0
Contra-
Ipsi-
Contra- 3D11
Contra- H2
APOE3-3D11
APOE3-H2
Contra- 4B12
Contra- F2
APOE4-4B12
APOE4-F2
D
E
APP
AT8
F
G
H
% APP Area
% AT8 Area
Number AT8+ puncta
APOE3 APOE4

Figure S7. *APOE3* and *APOE4* hAstrocytes differentially affect overall neuritic dystrophy and Tau pathology, related to Figure 7.

(A) Representative images of dystrophic neurites (APP, red) surrounding Aβ plaques (Thio, blue) in contralateral and ipsilateral cortices of AD chimeras with *APOE3* and *APOE4* hAstrocytes. Scale bars: 25 μm.

(B) Representative images of hyperphosphorylated Tau (AT8, green) surrounding Aβ plaques (Thio, blue). Scale bars: 50 μm.

(C) Percentage area occupied by AT8 around plaques in contra- and ipsilateral cortices (*APP PS1 Prkdc*$^{scid/scid}$; n=5 mice with *APOE3* and n=5 mice with *APOE4* astrocytes). Data are represented as mean ± SEM, paired t-test; ns = not significant, * = $p < 0.05$, ** = $p < 0.001$, *** = $p < 0.0001$.

(D) Immunofluorescence images of dystrophic neurites (APP, cyan) surrounding Aβ plaques in cortices of AD chimeras with *APOE3* and *APOE4* hAstrocytes. Images are taken from Figure 7. Scale bars: 25 μm.

(E) Representative images of hyperphosphorylated Tau (AT8, green) surrounding Aβ plaques in cortices of AD chimeras with *APOE3* and *APOE4* hAstrocytes. Images are taken from Figure 7. Scale bars: 50 μm.

(F) Percentage area occupied by APP positive dystrophic neurites around plaques in cortices of *APOE3* vs *APOE4* AD chimeras (*APP PS1 Prkdc*$^{scid/scid}$; n=5 mice with *APOE3* and n=5 mice with *APOE4* astrocytes).

(G) Percentage area occupied by AT8 around plaques (*APP PS1 Prkdc*$^{scid/scid}$; n=5 mice with *APOE3* and n=5 mice with *APOE4* hAstrocytes).

(H) Number of AT8 positive puncta around plaques (*APP PS1 Prkdc*$^{scid/scid}$; n=5 mice with *APOE3* and n=5 mice with *APOE4* astrocytes). Data are represented as mean ± SEM, unpaired t-test; ns = not significant, * = $p < 0.05$, ** = $p < 0.001$, *** = $p < 0.0001$.

Figure S8

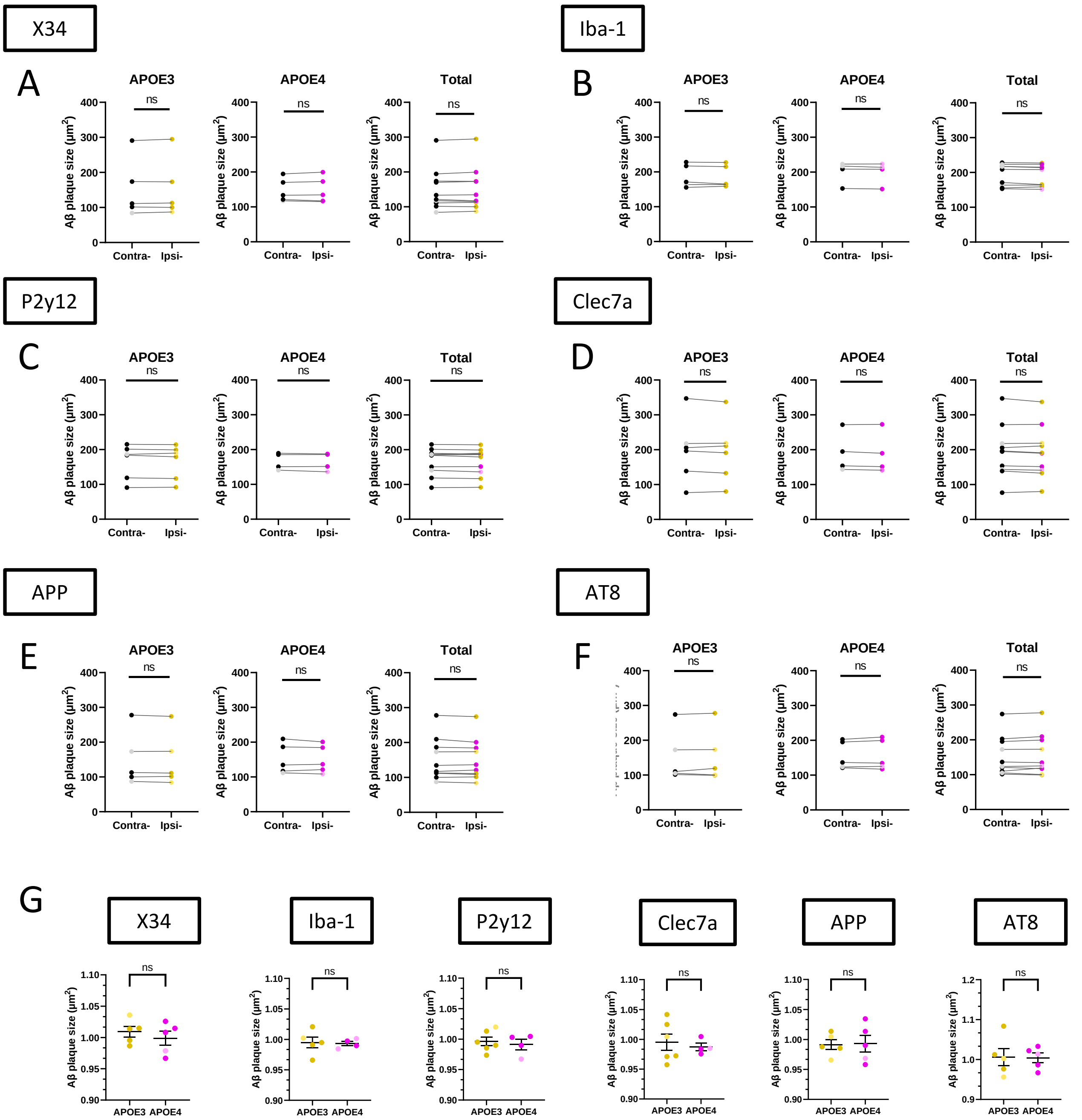

Figure S8. Aβ plaque size is consistent across all analyses performed in both the ipsilateral and contralateral regions, related to Figures 4, 5 and 7.

(A-F) Quantification of Aβ plaque size in all analyses, comparing the ipsilateral and contralateral regions for the plaque morphology with X34 marker and the Iba-1, Clec7a, P2y12, APP and the AT8 analyses around plaques. For each analysis, Aβ plaque size in contra- and ipsilateral cortical areas with *APOE3* and *APOE4* hAstrocytes is shown, followed by the combined data for all cases. Data are represented as mean ± SEM, paired t-test.

(G) Quantification of Aβ plaque size in all analyses, comparing AD chimeras with *APOE3* vs *APOE4* hAstrocytes. Data are represented as mean ± SEM, unpaired t-test; ns = not significant, * = p < 0.05, ** = p < 0.001, *** = p < 0.0001.

Table S1: Information of the hiPSC lines

hiPSC name	Ethnicity	Sex	Age of onset	Age at skin biopsy	Disease status (CDR at biopsy)	*APOE* genotype	Genetic modification
TCW1E33-1F1	Caucasian	F	64	72	AD (2)	E4/E4	E3/E3
TCW1E44-2C2	Caucasian	F	64	72	AD (2)	E4/E4	E4/E4
TCW2E33-2E3	Caucasian	M	77	80	AD (0.5)	E4/E4	E3/E3
TCW2E44-4B1	Caucasian	M	77	80	AD (0.5)	E4/E4	E4/E4
TCW2E33-3D11	Caucasian	M	77	80	AD (0.5)	E4/E4	E3/E3
TCW2E44-4B12	Caucasian	M	77	80	AD (0.5)	E4/E4	E4/E4
TCW3E33-H-2	Caucasian	M	80	83	AD (0.5)	E4/E4	E3/E3
TCW3E44-F-2	Caucasian	M	80	83	AD (0.5)	E4/E4	E4/E4

The table shows hiPSC name, patient ethnicity, sex, age of AD onset, age at skin biopsy, disease status (CDR at biopsy), original *APOE* genotype and genetic modification. F female, M male, AD Alzheimer's disease, *APOE* apolipoprotein, CDR clinical dementia rating, hiPSC human induced pluripotent stem cells. These cells were previously generated and characterized by TCW et al, 2022 [14] and Preman et al, 2021 [8]. Two sets of isogenic lines (2E3, 4B1 and 3D11, 4B12) are from the same donor.

Table S2. Assessment of integration efficiency of transplanted hiPSC-derived astrocytes.

hiPSC line	hiPSC name	Patient	*APOE* genotype	Genetic modification	Integration efficiency	Mice analyzed
1	TCW1E33 1F1	1	E4/E4	E3/E3	*	n = 5
2	TCW1E44 2C2	1	E4/E4	E4/E4	*	n = 5
3	TCW2E33 2E3	2	E4/E4	E3/E3	*	n = 7
4	TCW2E44 4B1	2	E4/E4	E4/E4	*	n = 7
5	TCW2E33 3D11	2	E4/E4	E3/E3	***	n = 5
6	TCW2E44 4B12	2	E4/E4	E4/E4	**	n = 5
7	TCW3E33 H-2	3	E4/E4	E3/E3	**	n = 6
8	TCW3E44 F-2	3	E4/E4	E4/E4	**	n = 8

* indicates less than 5,000 RFP+ cells, ** between 5,000 and 25,000 RFP+ cells, and *** between 25,000 and 200,000 RFP+ cells in the chimeric mouse brain.

Table S3. Details of hiPSC lines, number of mice and mouse model used on each experiment.

Figure	Experiment	Genotype mice & hiPSCs		hiPSC line injected	Nº mice used	Mouse model
Figure 2 C	Astrocytic h-APOE	WT	APOE3	3D11	4	*APP PS1* wt/wt *Prkdc*$^{scid/scid}$
				H2	3	
			APOE4	4B12	3	
				F2	3	
		AD	APOE3	3D11	5	*APP PS1* tg/wt *Prkdc*$^{scid/scid}$
				H2	2	
			APOE4	4B12	5	
				F2	2	
Figure 2 D,E,F	Extracellular h-APOE	WT	APOE3	3D11	3	*APP PS1* wt/wt *Prkdc*$^{scid/scid}$
				H2	2	
			APOE4	4B12	3	
				F2	2	
		AD	APOE3	3D11	4	*APP PS1* tg/wt *Prkdc*$^{scid/scid}$
				H2	1	
			APOE4	4B12	4	
				F2	1	
Figure 3 C,D	Plaque associated hAPOE	AD	APOE3	3D11	4	*APP PS1* tg/wt *Prkdc*$^{scid/scid}$
				H2	1	
			APOE4	4B12	4	
				F2	2	
Figure 4 B.<br>Figure S4B	Aβ burden (Thio)	AD	APOE3	3D11	5	*APP PS1* tg/wt *Prkdc*$^{scid/scid}$
				H2	2	
			APOE4	4B12	4	
				F2	2	
Figure 4 D.<br>Figure S4D	Aβ burden (6C3)	AD	APOE3	3D11	5	*APP PS1* tg/wt *Prkdc*$^{scid/scid}$
			APOE4	4B12	4	
				F2	1	
Figure 4 F,G<br>Figure S4 F,G	Plaque morphology (X34)	AD	APOE3	3D11	4	*APP PS1* tg/wt *Prkdc*$^{scid/scid}$
				H2	1	
			APOE4	4B12	4	
				F2	1	
Figure 5 B<br>Figure S6 E	Plaque-associated microglia (Iba-1)	AD	APOE3	3D11	4	*APP PS1* tg/wt *Prkdc*$^{scid/scid}$
				H2	1	
			APOE4	4B12	2	
				F2	2	
Figure 5 D,F<br>Figure S6 G,H	P2y12 and Clec7a positive microglia	AD	APOE3	3D11	5	*APP PS1* tg/wt *Prkdc*$^{scid/scid}$
				H2	1	
			APOE4	4B12	3	
				F2	1	
Figure 6 A-D	RNA-seq microglia	AD	APOE3	3D11	3	*App*$^{NL-G-F}$ *Rag2*$^{-/-}$
				H2	3	
			APOE4	4B12	4	
				F2	2	
Figure 6 F	Microglia coverage (Iba-1)	AD	APOE3	3D11	4	*APP PS1* tg/wt *Prkdc*$^{scid/scid}$
				H2	1	
			APOE4	4B12	2	
				F2	2	
Figure 6 H	Microglia expression of Cd68	AD	APOE3	3D11	2	*APP PS1* tg/wt *Prkdc*$^{scid/scid}$
				H2	1	
			APOE4	4B12	3	
				F2	1	
Figure 7 C,D<br>Figure S7 C, F-H	Tau pathology & neuritic dystrophy	AD	APOE3	3D11	3	*APP PS1* tg/wt *Prkdc*$^{scid/scid}$
				H2	2	
			APOE4	4B12	4	
				F2	1	
Figure S2 A,B	Soluble and insoluble hAPOE	AD	APOE3	3D11	8	*App*$^{NL-G-F}$ *Rag2*$^{-/-}$
				H2	6	
			APOE4	4B12	5	
				F2	6	
Figure S3 B.<br>Figure S4 I	Aβ burden (General)	AD	APOE3	3D11	5	*APP PS1* tg/wt *Prkdc*$^{scid/scid}$
				H2	1	
			APOE4	4B12	3	
				F2	2	
Figure S3 C,D.<br>Figure S4 J, K	Soluble and insoluble Aβ	WT	APOE3	3D11	6	*App*$^{hu/hu}$ *Rag2*$^{-/-}$
				H2	2	
			APOE4	4B12	5	
				F2	4	
		AD	APOE3	3D11	2	*App*$^{NL-G-F}$ *Rag2*$^{-/-}$
				H2	5	
			APOE4	4B12	1	
				F2	4	
Figure S6 B,C	Total microglia	AD	APOE3	3D11	5	*APP PS1* tg/wt *Prkdc*$^{scid/scid}$
				H2	1	
			APOE4	4B12	3	
				F2	2	

Table S4. Details of Human Cases

Case #	Sex	Age	*APOE* genotype	Braak stage	Thal stage	Neuropathological diagnosis
1	F	94	E3/E3	6	5	AD-VD
2	F	91	E3/E3	5	5	AD-VD
3	F	89	E3/E3	5	4	AD
4	F	89	E3/E3	5	4	AD
5	F	85	E3/E3	5	5	AD
6	F	84	E3/E3	6	5	AD
7	F	73	E4/E4	5	5	AD
8	M	75	E4/E4	5	5	AD-Lewy
9	F	86	E4/E4	5	4	AD-Lewy-VD
10	F	90	E4/E4	6	5	AD
11	F	90	E4/E4	6	5	AD
12	F	61	N/A	0	0	non-AD, control
13	F	85	N/A	0	0	non-AD, control
14	F	58	N/A	0	0	non-AD, control
15	M	43	N/A	0	0	non-AD, control

The table shows the human subjects used for histopathology analyses. Indicated are: the sex, the age in years, the *APOE* genotype, the Braak neurofibrillary tangle stage [56], the Thal stage representing the distribution of Aβ deposits [55], and the neuropathological diagnosis. F female, M male, AD Alzheimer's disease, AD-VD Alzheimer's disease plus signs of vascular disease, AD-Lewy Alzheimer's disease and Lewy Bodies.

Table S6. Summary of AD-related pathology and cellular changes associated with the presence of *APOE3* and *APOE4* hAstrocytes.

PATHOLOGY & RESPONSES	OBSERVATIONS IN DETAIL	APOE3 vs APOE4
**Amyloid pathology**	Total Thio+ plaque	↓
	Total Aβ staining	↓
	Plaque number	↓
	Average plaque size	=
	Plaque compactness	↑
**Tau pathology**	AT8 pathology around plaques	↓
**Neuritic dystrophy**	Dystrophic neurites	↓
**Microglia responses**	Total microgliosis	↓
	Microglia clustering around plaques	↓
	Microglia activation around plaques	= P2Y12
		↓ Clec7a
	Plaque coverage	=
	Microglia Cd68 expression	↓ Cd68
